# Fraxetin down-regulates polo-like kinase 4 (PLK4) to inhibit proliferation, migration and invasion of prostate cancer cells through the phosphatidylinositol 3-kinase (PI3K)/protein kinase B (Akt) pathway

**DOI:** 10.1080/21655979.2022.2054195

**Published:** 2022-04-07

**Authors:** Zheng Ma, Yanfang Sun, Weixing Peng

**Affiliations:** a Department of Urology, Suzhou Ninth Hospital Affiliated to Soochow University, Suzhou City, China; b Department of Ultrasound, Guanggu Branch of Wuhan Third Hospital, Wuhan City, China; c Department of Urology, Zhoushan Branch Shanghai Ruijin Hospital, Shanghai Jiaotong University, School of Medicine, Zhoushan City, China

**Keywords:** Fraxetin, prostate cancer, Polo-like kinase 4, PI3K/Akt

## Abstract

Fraxetin, a natural product isolated from herb *Cortex Fraxini*, has been demonstrated to exhibit anti-cancer effects on various cancers. The aim of this work is to investigate the anti-tumor effect of Fraxetin in prostate cancer and the potential mechanisms. In this study, the prostatic epithelial cell RWPE-1 and prostate cancer cell DU145 were exposed to Fraxetin (10, 20, 40, and 80 μM) to detect the changes in cell viability using cell counting kit-8 (CCK-8) assay. Fraxetin (10, 20, and 40 μM) was utilized to treat DU145 cell, then the changes in cell proliferation, apoptosis, migration, and invasion were assessed. Western blot assay was employed to detect the expression of proteins that participate in the above cellular processes as well as Polo-like kinase 4 (PLK4), phosphatidylinositol 3-kinase (PI3K). In addition to 40 μM Fraxetin treatment, DU145 cells were overexpressed with PLK4, and then the above experiments were repeated. Results revealed that Fraxetin markedly decreased DU145 cell viability, but didn’t affect the cell viability of RWPE-1. Fraxetin suppressed cell proliferation, migration, invasion, and induced apoptosis of DU145 cells in a concentration-dependent manner. Furthermore, the expression of PLK4 and phosphorylated PI3K and protein kinase B (Akt) were reduced upon Fraxetin treatment. Finally, PLK4 overexpression significantly reversed all the effects of Fraxetin on DU145 cells. Collectively, Fraxetin acted as a cancer suppressor in prostate cancer through inhibiting PLK4 expression thereby inactivating PI3K/Akt signaling.

## Graphical Abstract

**Figure:**
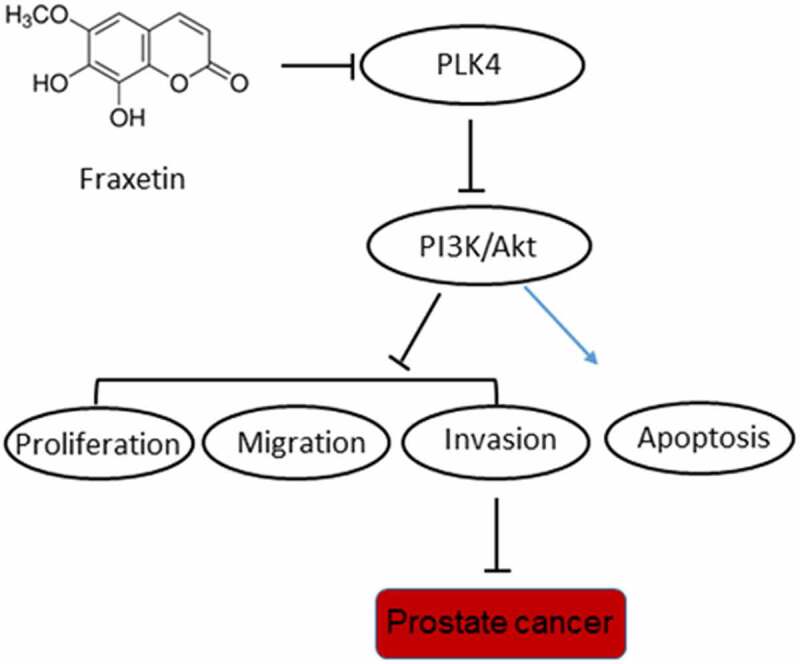

## Introduction

Fraxetin is a natural coumarin compound that is extracted from the Chinese herb *Cortex Fraxini*. Modern pharmacological studies have supported that Fraxetin has various pharmacological properties, such as antibacterial and anti-inflammatory, inhibiting oxidative stress, neuroprotection, tumor suppression, and immunomodulation effects [1]. Accumulating studies have reported the anti-cancer effects of Fraxetin on a variety of tumors, such as liver cancer [2], breast cancer [3], colon adenocarcinoma [4], pancreatic cancer [5], and glioma [6]. These studies illustrated the powerful potential of Fraxetin in anti-cancer, but whether Fraxetin exhibited anti-cancer effect on prostate cancer remain to be clarified.

Polo-like kinase 4 (PLK4) is a serine/threonine protein kinase that mediates centriole duplication, the dysregulation of which leads to tumorigenesis [7]. PLK4 can also regulate cancer cells invasion and metastasis via mediating the events of actin cytoskeleton [8]. Hence, PLK4 has been considered as a therapeutic target for treating various cancer [9]. For example, inhibition of PLK4 activity has been implicated to be associated with colorectal cancer suppression [10]. Besides, a novel PLK4 inhibitor, YLZ-F5, was reported to restrain ovarian tumor growth through promoting apoptosis and mitotic defects [11]. Notably, a previous study reported that CAND1 promoted PLK4-regulated centriole over-duplication in prostate cancer [12], indicating the potential role of PLK4 in prostate cancer.

Recently, PLK4 was illustrated to regulate the phosphatidylinositol 3-kinase (PI3K)/protein kinase B (Akt) signaling pathway to play its role in cancers [13,14]. PLK4 is a target of Fraxetin in Swiss Target Prediction (http://www.swisstargetprediction.ch/) database. Therefore, we hypothesized that Fraxetin might exerted anti-cancer effect on prostate cancer through regulating PLK4 expression, subsequently affecting PI3K/Akt signaling.

The purpose of this study is to confirm the effects of Fraxetin on the proliferation, apoptosis, migration, and invasion of prostate cancer cells. The latent regulatory mechanism of Fraxetin related to PLK4 and PI3K/Akt signaling in prostate cancer progression was explored. Our findings might offer a novel candidate drug for the therapeutic strategy of prostate cancer.

## Materials and methods

### Drugs

Fraxetin (cat no: 574–84-5, purity ≥98%) was purchased from Solarbio (Beijing, China) and dissolved in dimethyl sulfoxide at a concentration of 100 mM.

### Cell culture and treatment

The human normal prostatic epithelial cell RWPE-1 and prostate cancer cell DU145 provided by American Type Culture Collection (ATCC, USA) were maintained in Roswell Park Memorial Institute (RPMI)-1640 medium (Gibco, USA) containing 10% fetal bovine serum (FBS, Hyclone, USA), 1% penicillin and streptomycin, at 37°C with 5% CO_2_.

### Cell transfection

DU145 cell were seeded into 6-well plates (2x10^5^ cells/well) and cultured at 37°C until they reached 80% confluence. For PLK4 overexpression, the human PLK4 gene was cloned into a pCMV6-AC-GFP destination vector, namely overexpression (Ov)-PLK4. The empty pCMV6-AC-GFP vector was used as negative control (Ov-NC). These constructs were transfected into DU145 cells using lipofectamine 2000 (Invitrogen, USA) according to the manufacture’s protocol. At 24 h post-transfection, the transfection efficiency was determined by using reverse transcription-quantitative PCR (RT-qPCR) and western blot analysis.

### Cell counting kit-8 (CCK-8)

The effect of Fraxetin on RWPE-1 and DU145 cell viability was evaluated with a CCK-8 Assay Kit (Dojindo, Tokyo, Japan). In brief, 4 × 10^3^ cells per well were seeded into 96-well plates, followed by being treated with Fraxetin (0, 10, 20, 40, and 80 μM) for different hours (24, 48, and 72 h) [15]. Subsequently, cells were incubated with 10 µl CCK-8 solution for 2 h and the absorbance at 450 nm was analyzed via a microplate reader (Bio-Rad, Hercules, CA, USA).

### Colony formation assay

DU145 cells (1000 cells/well) were plated in 6-well plates, then exposed to different concentrations of Fraxetin (10, 20, and 40 µM). After 24 h, the culture medium was replaced with RPMI-1640 medium and cultured for another 14 days. Colonies were fixed with methanol for 15 min, stained with crystal violet for 15 min and finally counted and photographed using a light microscope (Olympus Corporation).

### Terminal-deoxynucleoitidyl transferase mediated nick end labeling (Tunel) staining

The apoptosis of DU145 cells was visualized using Tunel staining. After treatment with Fraxetin (0, 10, 20, or 40 μM) for 48 h, cells were fixed by 4% paraformaldehyde for 0.5 h, incubated with hydrogen peroxide for 15 min, and then treated with Triton X100 for 5 min. After being treated with the Tunel reaction system (Beyotime, China) at room temperature for 2 h in the dark, the cells were stained with 4’,6-diamidino-2-phenylindole (DAPI) for nucleus staining and images were obtained using a fluorescence microscope (magnification, x200; Olympus Corporation). The tunel-positive cells were counted by ImageJ software (National Institutes of Health).

### Western blotting

Total protein from DU145 cells was isolated using the radioimmunoprecipitation (RIPA) buffer (Beyotime), and with the application of bicinchoninic acid (BCA) kit (Beyotime), the protein concentration was determined. Protein samples were then separated by 12% sodium dodecyl sulfate-polyacrylamide gel electrophoresis (SDS-PAGE), followed by being transferred to a polyvinylidene fluoride (PVDF) membrane. After exposure to skimmed milk for 2 h at 37°C, the membranes were incubated with primary antibodies against glyceraldehyde-phosphate dehydrogenase (GAPDH; #5174 T, 1:1000 dilution; Cell Signaling Technology), B-cell lymphoma 2 (Bcl-2; #4223 T, 1:1000 dilution; Cell Signaling Technology), BCL2-associated X protein (Bax; #5023 T, 1:1000 dilution; Cell Signaling Technology), caspase 3 (#9662S, 1:1000 dilution; Cell Signaling Technology), cleaved-caspase 3 (#9664 T, 1:1000 dilution; Cell Signaling Technology), poly ADP-ribose polymerase (PARP; #9532 T, 1:1000 dilution; Cell Signaling Technology), cleaved-PARP (#5625 T, 1:1000 dilution; Cell Signaling Technology), E-cadherin (#3195S, 1:1000 dilution; Cell Signaling Technology), N-cadherin (#13,116 T, 1:1000 dilution; Cell Signaling Technology), Vimentin (#5741 T, 1:1000 dilution; Cell Signaling Technology), PI3K (ab227204, 1:1000 dilution; Abcam), phosphorylated (p)-PI3K (ab278545, 1:1000 dilution; Abcam), Akt (#9272S, 1:1000 dilution; Cell Signaling Technology), and p-Akt (ab38449, 1:1000 dilution; Abcam), overnight at 4°C. The goat anti-rabbit horseradish peroxidase (HRP)-conjugated secondary antibody (#7074S; 1:3000 dilution; Cell Signaling Technology) was used at room temperature for 2 h. Enhanced chemiluminescence (ECL) solution (Bio-Rad Laboratories, USA) was utilized to develop the blots and blots were visualized with a gel imaging system (Bio-Rad). The relative protein expression was calculated using GAPDH as the loading control.

### Wound-healing assay

Cells were seeded in 6-well plates at a density of 1 × 10^4^ cells/well and cultured at 37°C until 90% confluence. Afterward, a crystal pipette tip was employed to create a linear gap between the cells. The cells were washed with phosphate buffer solution (PBS) three times before the addition of new serum-free medium containing a series concentration of Fraxetin (0, 10, 20, and 40 μM) for another 48 h [6]. Finally, images of the culture area were captured under a light microscope (magnification, x100; Olympus Corporation).

### Transwell assay

Transwell assay was employed to detect cell invasion ability. The chambers were coated with Matrigel (Corning), and then, DU145 cells were diluted in serum-free RPMI-1640 containing Fraxetin (0, 10, 20, or 40 μM) and then seeded into the upper chamber. The lower chamber was filled with RPMI-1640 containing 10% FBS. After incubation at 37°C for 24 h, the invaded cells on the lower surface were fixed with formalin and stained with 0.5% crystal violet (Sigma). Finally, images of the invaded cells were captured under a light microscope (magnification, x100; Olympus Corporation).

### RT-qPCR

After being treated with Fraxetin for 48 h, RNA from cells was extracted using TRIZOL buffer (Thermo Fisher Scientific). Reverse transcription was carried out using ReverTra AceqPCR RT Master Mix (Toyobo, Japan). With the application of SYBR Green Kit (Sigma-Aldrich), qPCR was performed on a StepOnePlus Real-Time PCR system (Applied Biosystems, USA). The relative gene expression was analyzed using the 2^−ΔΔCT^ method after normalization to GAPDH [16]. Primers used in this experiments were as follow: PLK4, forward 5’-CACTGAATTCCATGGCGACCTGCATCGGGG-3’, reverse: 5’-CTGCGGTACCTTATCAATGAAAATTAGGAG-3’; GAPDH: forward 5’-AATGGGCAGCCGTTAGGAAA-3’, reverse 5’-GCGCCCAATACGACCAAATC-3’.

### Statistical analysis

All experiments were performed in three independent replicates. Data are analyzed using GraphPad Prism 8.0 and presented as mean ± standard deviation. A one-way analysis of variance (ANOVA) followed by Tukey’s test was used to analyze the statistical differences, which was thought significant when P value less than 0.05.

## Results

### Fraxetin restrains proliferation and promotes apoptosis of DU145 prostate cancer cells

**Figure 1.**
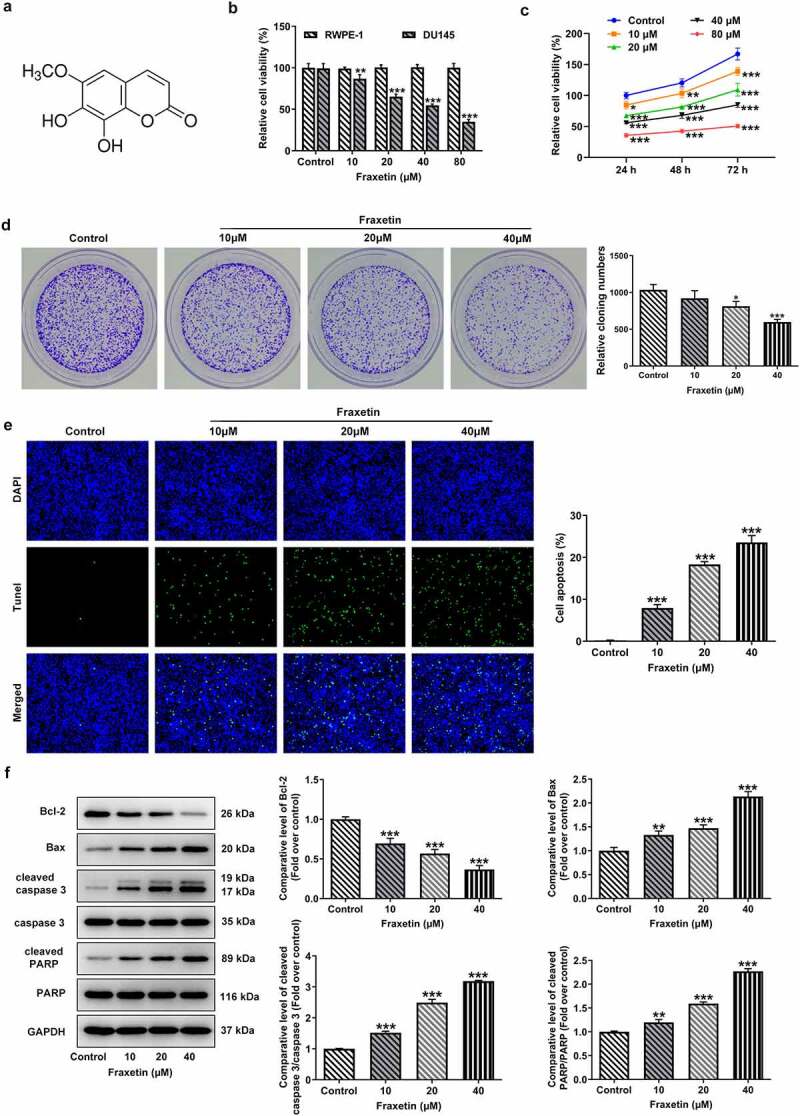
Fraxetin inhibits proliferation and induces apoptosis of DU145 prostate cancer cells. (a) the chemical structure of Fraxetin. (b) RWPE-1 and DU145 cells were treated with 0, 10, 20, 40 and 80 μM Fraxetin for 48 h, then cell viability was assessed using CCK-8 assay. (c) DU145 cell was treated with to 0, 10, 20, 40 and 80 μM Fraxetin for 24, 48 and 72 h, then cell viability was detected using CCK-8 assay. (d) colony formation assay was used to detect proliferation of DU145 cells that in the presence of 0, 10, 20 and 40 μM Fraxetin. E, the apoptosis of DU145 cell was observed by Tunel staining (magnification, x200). F, the protein expression of Bcl-2, Bax, cleaved-caspase 3/caspase 3 and cleaved PARP/PARP in DU145 was detected by western blot assay. *P < 0.05, **P < 0.01 and ***P < 0.001 vs Control.

As a natural coumarin compound that is extracted from the Chinese herb *Cortex Fraxini*, Fraxetin has been reported exert tumor suppression effects in several human cancers [3,4]. This study was the first to explore whether Fraxetin exhibited anti-cancer effect on prostate cancer. Figure 1a illustrated the chemical structure of Fraxetin. Firstly, the human normal prostatic epithelial cell RWPE-1 and prostate cancer cell DU145 were subjected to different concentrations of Fraxetin (0, 10, 20, 40, and 80 μM) for 48 h, then cell viability was measured. As shown in Figure 1b, Fraxetin exerted no obvious effect on RWPE-1 viability but remarkably reduced DU145 cell viability. This result not only ensured the security of Fraxetin on normal prostatic epithelial cell but also indicated the anti-cancer potency of Fraxetin in prostate cancer.

To explore the effect of Fraxetin on prostate cancer cells proliferation, CCK-8 and colony formation assays were carried out in the subsequent experiments. Results showed that different concentrations of Fraxetin (10, 20, 40, and 80 μM) significantly inhibited cell viability at 24, 48, and 72 h post-treatment (Figure 1c). Fraxetin at 10, 20, and 40 μM was chosen for next experiments considering that 80 μM Fraxetin resulted in more than 50% decrease in DU145 cell viability. The colony number of DU145 prostate cancer cells was obviously decreased upon 10, 20, and 40 μM Fraxetin treatment, indicating the negative effect of Fraxetin on prostate cancer cells proliferation (Figure 1d).

Then, the changes in cell apoptosis under Fraxetin treatment were evaluated. As shown in Figure 1e, Fraxetin caused marked increase in the ratio of apoptotic prostate cancer cells. Similarly, Fraxetin changed the expression of proteins that regulate cell apoptosis, as evidence by down-regulated Bcl-2 expression, but up-regulated expression of pro-apoptotic proteins including Bax, cleaved caspase-3 and cleaved PARP, in response to Fraxetin treatment (Figure 1f). These data illustrated the inducible effect of Fraxetin on prostate cancer cells apoptosis.

### Fraxetin inhibits migration and invasion of DU145 prostate cancer cells

**Figure 2.**
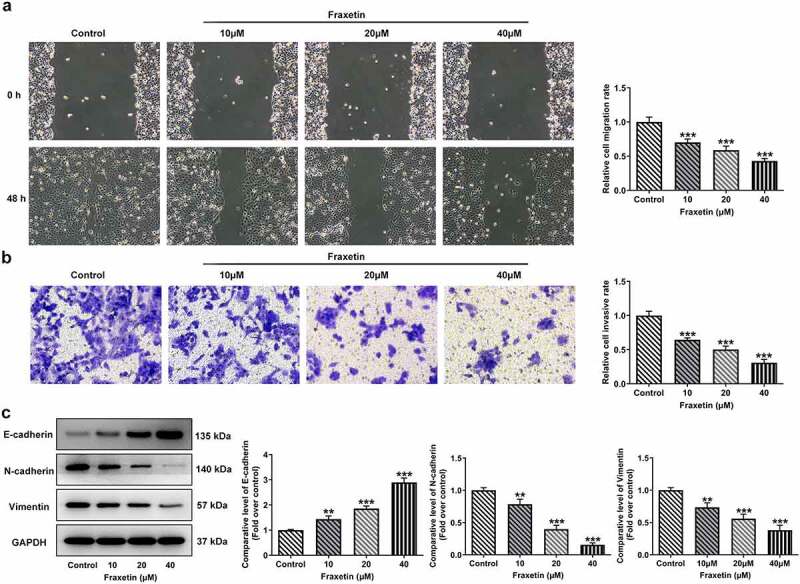
Fraxetin inhibits migration and invasion of DU145 prostate cancer cells. DU145 cells were treated with 0, 10, 20 and 40 μM Fraxetin for 48 h, then (a) cell migration was observed by wound-healing assay (magnification, x100); (b) cell invasion was measured by transwell assay (magnification, x100); (c) the protein expression was detected by western blot assay. **P < 0.01 and ***P < 0.001 vs Control.

To study the effects of Fraxetin on the migration and invasion of prostate cancer cells, wound-healing assay, and transwell assay were conducted in the following experiments. Results revealed that, Fraxetin (10, 20, and 40 μM) treatment resulted in a significant decrease in cell migration distance compared with control group (Figure 2a). Similarly, the ratio of invaded cells was markedly decreased by Fraxetin treatment (Figure 2b). Furthermore, in comparison to control DU145 cells, cells that exposed to different concentrations of Fraxetin (10, 20, and 40 μM) exhibited increased expression of E-cadherin, and decreased expression of N-cadherin and Vimentin (Figure 2c). These data revealed the suppression of prostate cancer cells migration and invasion caused by Fraxetin.

### Fraxetin down-regulates PLK4 expression and inhibits PI3K/Akt activation in DU145 prostate cancer cells

**Figure 3.**
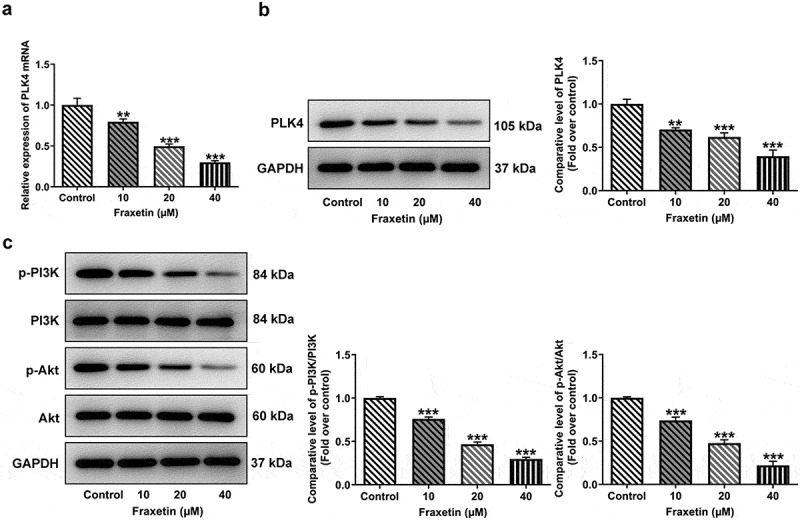
Fraxetin down-regulates the expression of PLK4 and phosphorylated PI3K/Akt in DU145 prostate cancer cells. DU145 cells were treated with 0, 10, 20 and 40 μM Fraxetin for 48 h, then (a-b) PLK expression was detected by RT-qPCR and western blot assays; (c) protein expression of phosphorylated PI3K and Akt was measured by western blotting. **P < 0.01 and ***P < 0.001 vs Control.

To disclose the possible mechanisms underlying the actions of Fraxetin in prostate cancer, we assessed the effects of Fraxetin on PLK4 expression in DU145 cells, considering that PLK4 is predicted to be a target of Fraxetin in Swiss Target Prediction (http://www.swisstargetprediction.ch/) database. It was found that DU145 cells that exposed to Fraxetin had lower expression of PLK4, when compared with control cells (Figure 3a-b). PLK4 can regulate the PI3K/Akt signaling pathway to play its role in cancers [13,14]. We also detected the expression of PI3K/Akt in DU145 cells before and after Fraxetin treatment. Figure 3c revealed that Fraxetin (10, 20, and 40 μM) reduced the phosphorylation of PI3K and Akt, suggesting the inhibition of Fraxetin on PI3K/Akt signaling activation. It was hence hypothesized that Fraxetin may exhibit its inhibitory effect on prostate cancer via down-regulating PLK4 expression thereby inactivating PI3K/Akt signaling.

### PLK4 reverses the effect of Fraxetin on DU145 prostate cancer cells

**Figure 4.**
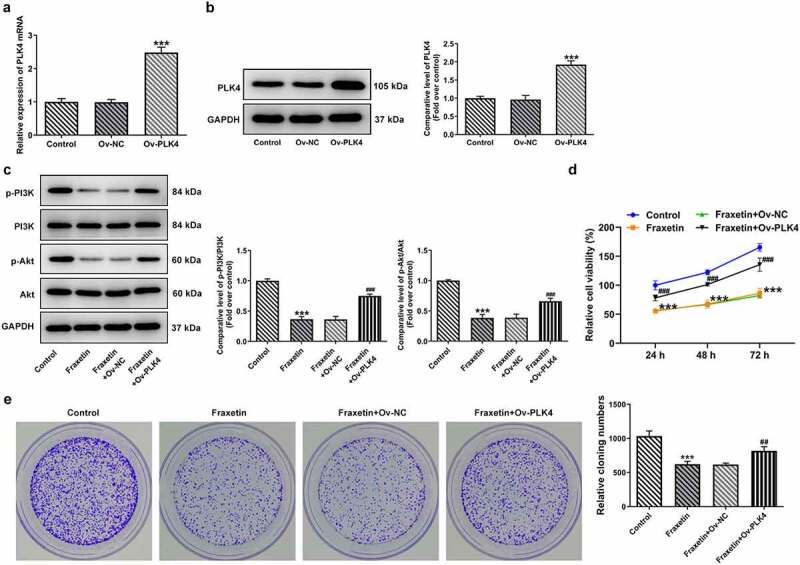
PLK4 overexpression reverses the effect of Fraxetin on PI3K/Akt expression and cell proliferation in DU145 prostate cancer cells. (a-b) DU145 cell was overexpressed with PLK4 via transfection of Ov-PLK4, the transfection efficiency was validated by RT-qPCR and western blotting. ***P < 0.001 vs Ov-NC. C-E, control DU145 cells or cells that transfected with indicated vectors were exposed to 40 μM Fraxetin or not, then (c) the expression of phosphorylated PI3K and Akt was detected by western blotting; (d) cell viability at 24, 48 and 72 h post-treatment was measured by CCK-8 assay; (e) cell proliferation was observed by colony formation assay. ***P < 0.001 vs Control; ^##^P < 0.01, ^###^P < 0.001 vs Fraxetin+Ov-NC.

**Figure 5.**
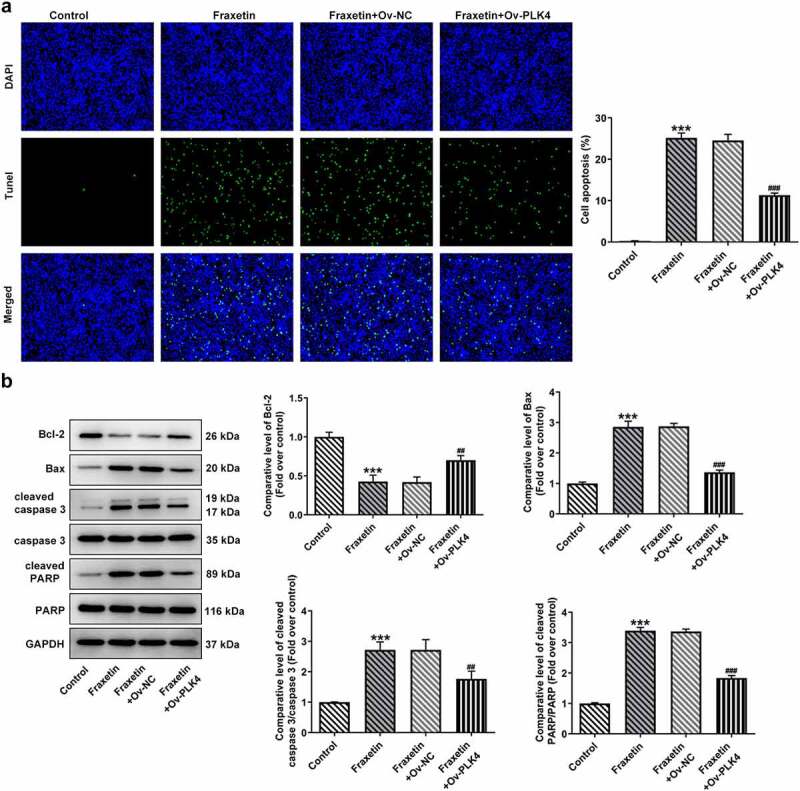
PLK4 overexpression reverses the effect of Fraxetin on apoptosis of DU145 prostate cancer cells. Control or transfected DU145 cells were exposed to 40 μM Fraxetin or not, then (a) cell apoptosis was explored via Tunel staining (magnification, x200); (b) the protein expression of Bcl-2, Bax, cleaved-caspase 3/caspase 3 and cleaved PARP/PARP was evaluated by western blot assay. ***P < 0.001 vs Control; ^##^P < 0.01 and ^###^P < 0.001 vs Fraxetin+Ov-NC.

**Figure 6.**
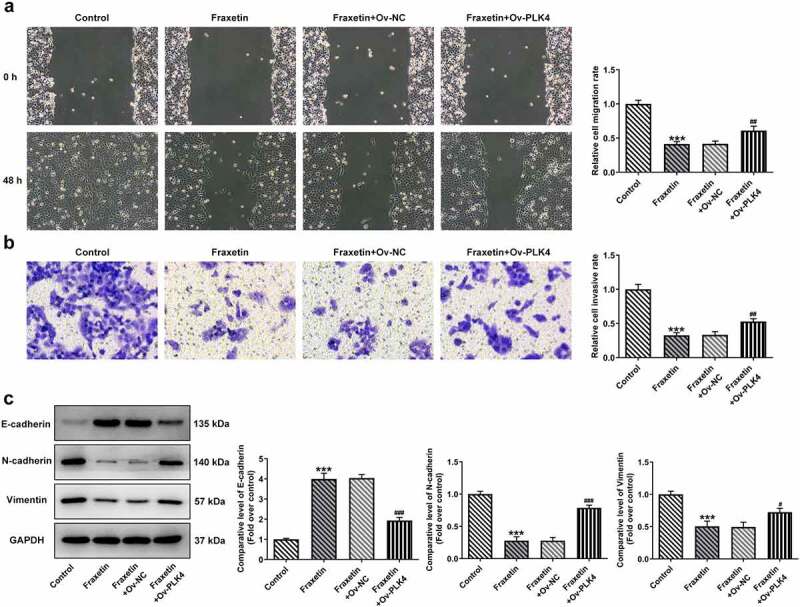
PLK4 overexpression reverses the effect of Fraxetin on migration and invasion of DU145 prostate cancer cells. Control DU145 cells or cells that transfected with indicated vectors were exposed to 40 μM Fraxetin or not, then (a) cell migration was observed by wound-healing assay (magnification, x100); (b) cell invasion was measured by transwell assay (magnification, x100); (c) the protein expression was detected by western blot assay. ***P < 0.001 vs Control; ^#^P < 0.05, ^##^P < 0.01 and ^###^P < 0.001 vs Fraxetin+Ov-NC.

To further validate the above speculation, DU145 cells were overexpressed with PLK4 by transfection with Ov-PLK4, and Ov-NC was used as the negative control (Figure 4a-b). Fraxetin (40 μM) was exposed to control DU145 cells or cells transfected with Ov-PLK4 or Ov-NC. Then, the expression of PI3K/Akt was measured, and results showed that Fraxetin inhibited the expression of p-PI3K and p-Akt compared with control cells, but PLK4 overexpression enhanced p-PI3K and p-Akt expressions compared with Ov-NC, in the presence of Fraxetin (Figure 4c). These results revealed that the Fraxetin-mediated inactivation of PI3K/Akt signaling was reversed by PLK4 overexpression. In the end, the changes in DU145 cells proliferation, apoptosis, migration, and invasion, caused by PLK4 overexpression were evaluated. Figure 4d demonstrated that 40 μM Fraxetin treatment reduced cell viability at 24, 48, and 72 h post-treatment, but additional PLK4 overexpression remarkably increased cell viability when compared with Fraxetin + Ov-NC group. At the same time, DU145 in Ov-PLK4 group showed increased number of colonies than that in Ov-NC group, in the presence of Fraxetin treatment (Figure 4e). These data demonstrated that PLK4 overexpression reversed the increased cell proliferation upon Fraxetin treatment. PLK4 overexpression also significantly restrained the actions of Fraxetin on apoptosis (Figure 5a-b), cell migration, and invasion (Figure 6a-c). Thus, all the effects of Fraxetin on prostate cancer cells could be blocked by PLK4 upregulation.

## Discussion

Prostate cancer is one of the common malignant tumors in men in males over the age of 50, which has become a public health issue that is highly valued worldwide [17,18]. Previous studies showed that approximately 10% of newly diagnosed patients had metastatic lesions, and 5% eventually developed metastatic after surgery [19,20]. Despite the great progress in treatment efficiency, the long-term survival of prostate cancer patients is still poor [21]. The underlying mechanism of the initiation and development of prostate cancer is still not fully understood. In the current study, we demonstrated the promising anticancer activity of Fraxetin in prostate cancer. Although previous studies have reported the anticancer effects of Fraxetin on multiple types of cancers, this is the first study that showed its potency in inhibiting prostate cancer. Importantly, we found that the anti-cancer effect of Fraxetin on prostate cancer may depend on inhibiting PLK4 expression thereby inactivating PI3K/Akt signaling. Our results implied Fraxetin as a promising candidate for prostate cancer therapy.

Studies have shown that Fraxetin exerted it anti-cancer effect via suppressing the malignant progression of cancer cells. For example, Fraxetin inhibits the proliferation of human liver cancer cell lines Huh7 and Hep3B as well as induces apoptosis through mitochondrial dysfunction [2]. Fraxetin also inhibits MCF-7 breast cancer cells proliferation of and induces cell apoptosis [3]. A recent study reported that Fraxetin inhibits proliferation and metastasis of glioma cells by inactivating JAK2/STAT3 signaling [6]. Tumor invasion and metastasis remain the major causes of deaths in prostate cancer patients [22]. Here, we showed that Fraxetin had no obvious effect on human normal prostatic epithelial cell RWPE-1, but significantly impaired cell viability of prostate cancer cell DU145 in a concentration-dependent manner. These data illustrated the hurtlessness of Fraxetin on normal prostatic cells, as well as indicated its potential in alleviating prostate cancer. Further functional analysis revealed that Fraxetin suppressed proliferation, promoted apoptosis, inhibited migration, and invasion of prostate cancer cells, suggesting the inhibitory effect of Fraxetin on inhibiting prostate cancer aggravation in vitro.

PLK4 is a member of the Polo-like kinases (PLKs) family, which is a family of serine/threonine kinases that play a critical role in cell cycle regulation and cellular responses under stress [23]. PLK4 is regarded as a crucial regulator of centriole duplication and mitotic progression, the dysregulation of it results in loss of centrosome numeral integrity, which leads to genomic instability and triggers tumorigenesis [9]. Studies suggest increased expression of PLK4 in various cancers and its association with tumorigenesis, cancer metastasis, resistance to chemotherapy and poor cancer prognosis [^24–26^]. Thus, PLK4 has been proposed as a therapeutic target for anticancer drug development. Fraxetin was shown to target PLK4 in Swiss Target Prediction (http://www.swisstargetprediction.ch/) database, and this study found that Fraxetin down-regulated PLK4 expression in a concentration-dependent manner. Several studies have shown that PLK4 inhibition may lead to cancer cell death [10,27]. We therefore speculated that Fraxetin may exert its anti-cancer effect through down-regulating PLK4 expression. Further results showed that Fraxetin also inhibited the phosphorylation of PI3K and Akt. The PI3K/Akt signaling pathway is crucial to many aspects of cell proliferation and survival, thus playing an important role in tumorigenesis [28]. Oncogenic activation of the PI3K/Akt pathway is common in prostate cancer, which can promote cancer progression and chemoresistance [29]. These data suggested that it is inactivation of PI3K/Akt that ultimately led to inhibition of prostate cancer cells aggravation.

In addition, PLK4 overexpression not only recovered the expression of phosphorylated PI3K and Akt but also blocked the effect of Fraxetin on prostate cancer cells proliferation, apoptosis, migration, and invasion, revealing the crucial role of PLK4 in Fraxetin-mediated PI3K/Akt inactivation and prostate cancer inhibition. Although it has been reported that PLK4 downregulation may contribute to cancer cell death, but the possible mechanisms are unknown. In the present study, we discovered that the decrease in p-PI3K (PI3K p85 subunit) and p-Akt expressions caused by Fraxetin was significantly recovered by PLK4 overexpression. In neuroblastoma cells, PLK4 has been found to mediate epithelial–mesenchymal transition tumorigenesis and cisplatin resistance via PI3K/Akt signaling pathway [14,30]. Consistently, our results illustrated that PLK4 play a role in cancer cells through regulating PI3K/Akt activation. However, only one prostate cancer cell line was used to demonstrate the inhibitory effects and mechanisms of Fraxetin on prostate cancer in this study, which is a limitation of the present study. The specific-binding mechanism between fraxetin and PLK4 as well as the verification of the current findings on other prostate cancer cell lines are the aim our following investigations.

## Conclusion

Collectively, the present study for the first time showed that Fraxetin inhibited prostate cancer cells proliferation, migration, and invasion. Mechanistically, Fraxetin down-regulated PLK4 expression thereby inactivating PI3K/Akt signaling pathway. Our results may provide a theoretical and experimental basis for the exploiting novel candidates for prostate cancer.

## Supplementary Material

Supplemental Material

## Data Availability

All data included in this study are available upon request through contact with the corresponding author.
