# Supplementary figures and images for: Fraxetin down-regulates polo-like kinase 4 (PLK4) to inhibit proliferation, migration and invasion of prostate cancer cells through the phosphatidylinositol 3-kinase (PI3K)/protein kinase B (Akt) pathway
Source: Bioengineered. 2022 Apr 7;13(4):9345–56. doi: 10.1080/21655979.2022.2054195 (PMC9161838; doi:10.1080/21655979.2022.2054195)

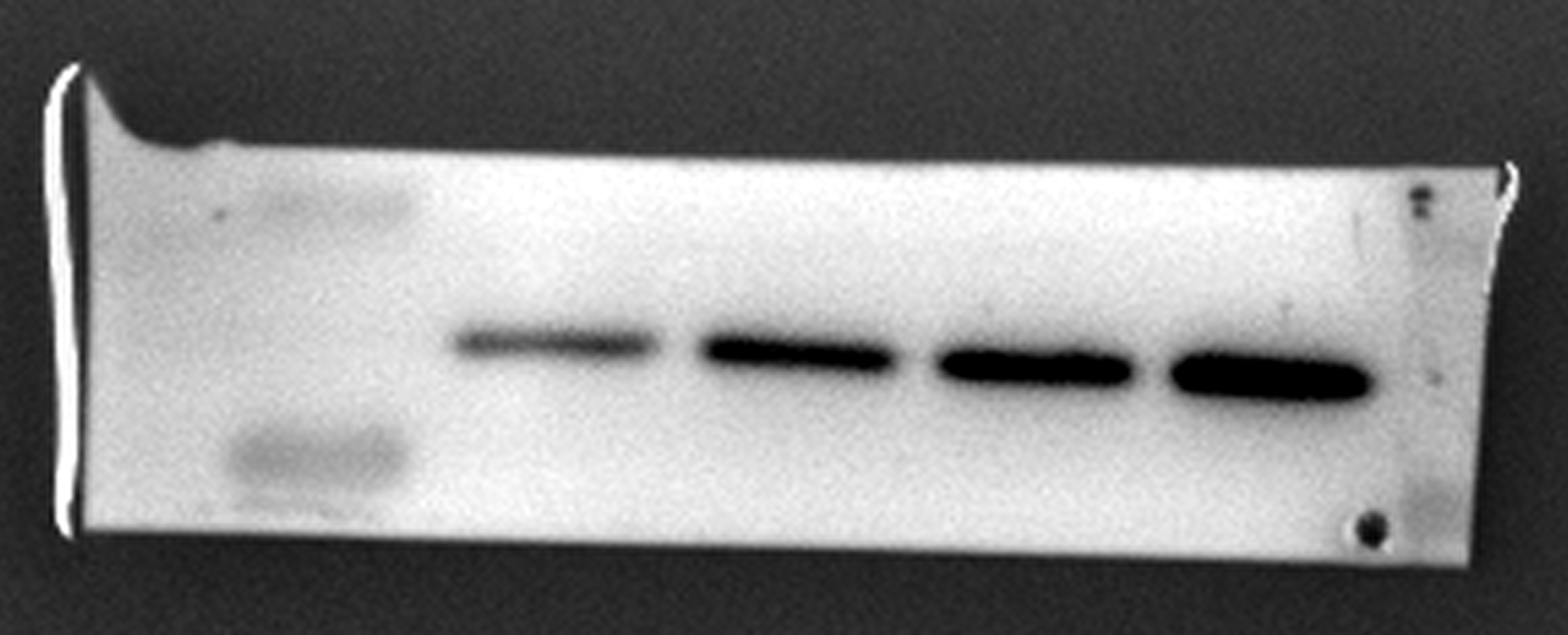

Supplement: Supplemental Material [file KBIE_A_2054195_SM0485.zip › supplementary/Figure1F_Bax.tif]

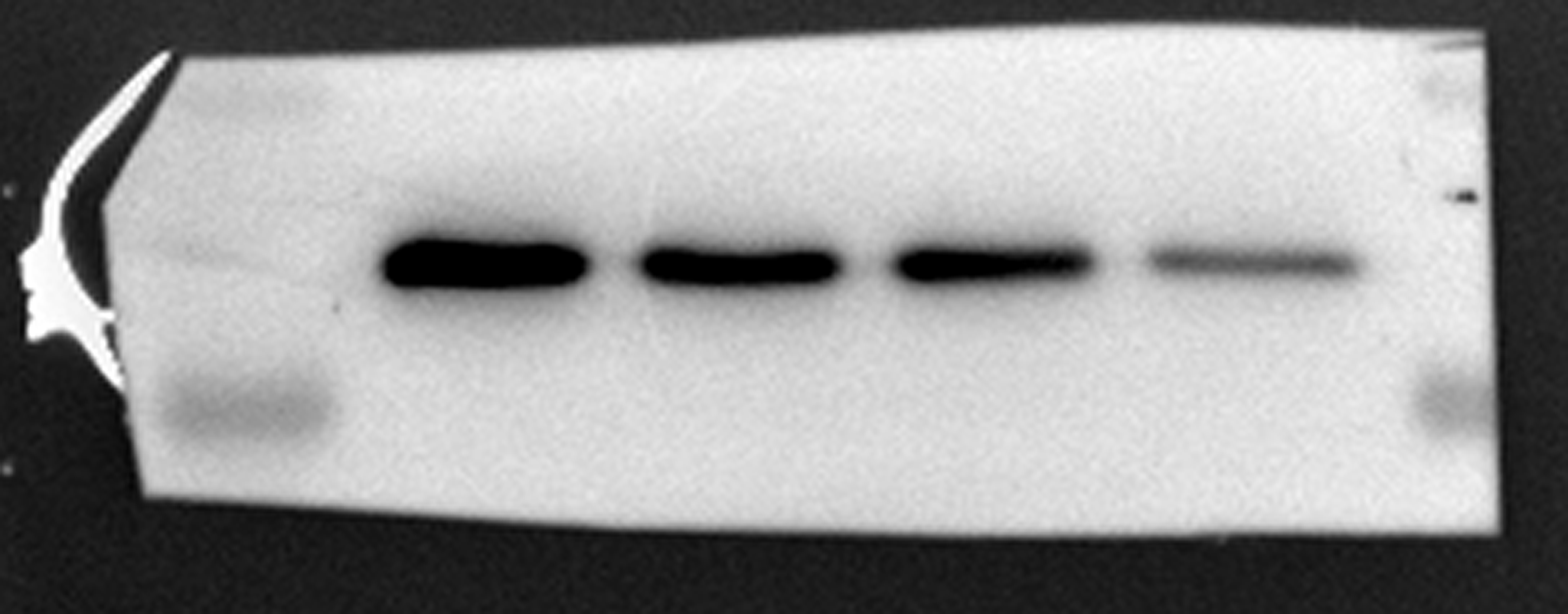

Supplement: Supplemental Material [file KBIE_A_2054195_SM0485.zip › supplementary/Figure1F_Bcl_2.tif]

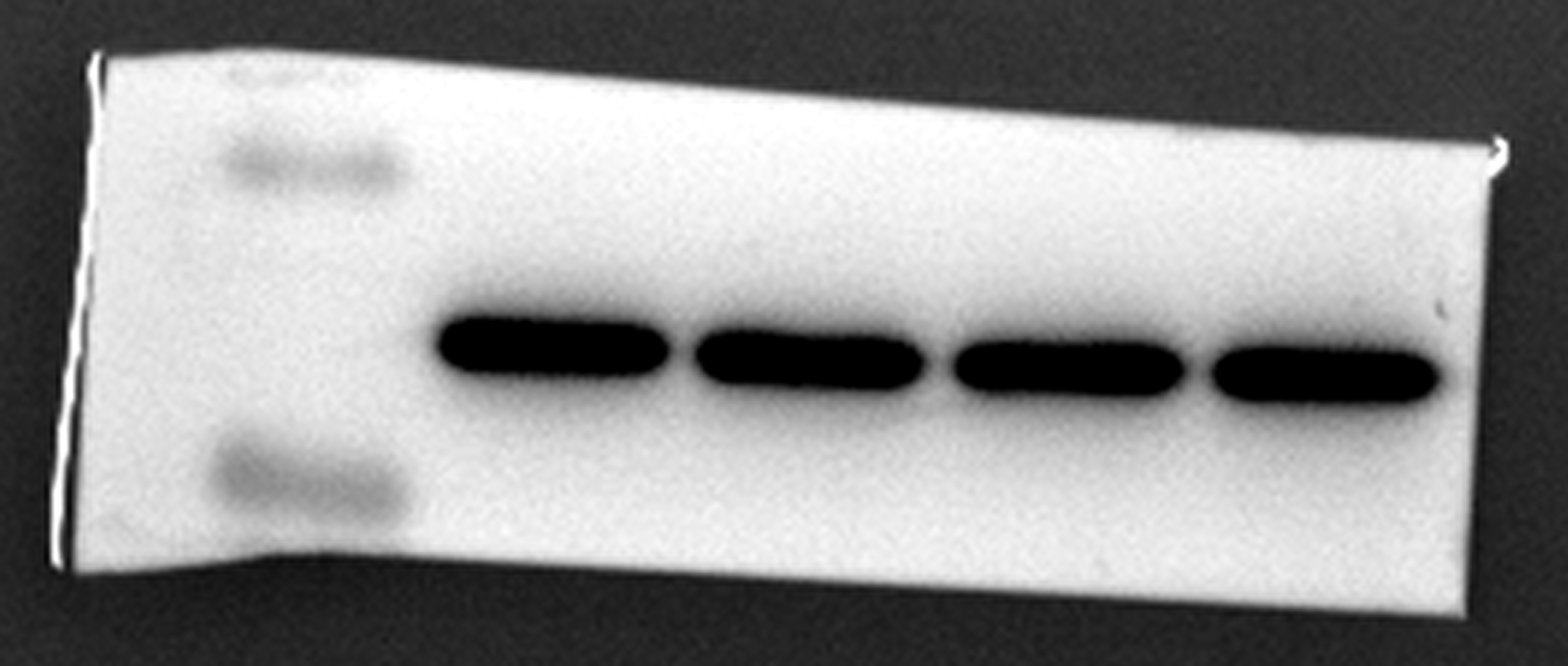

Supplement: Supplemental Material [file KBIE_A_2054195_SM0485.zip › supplementary/Figure1F_caspase3.tif]

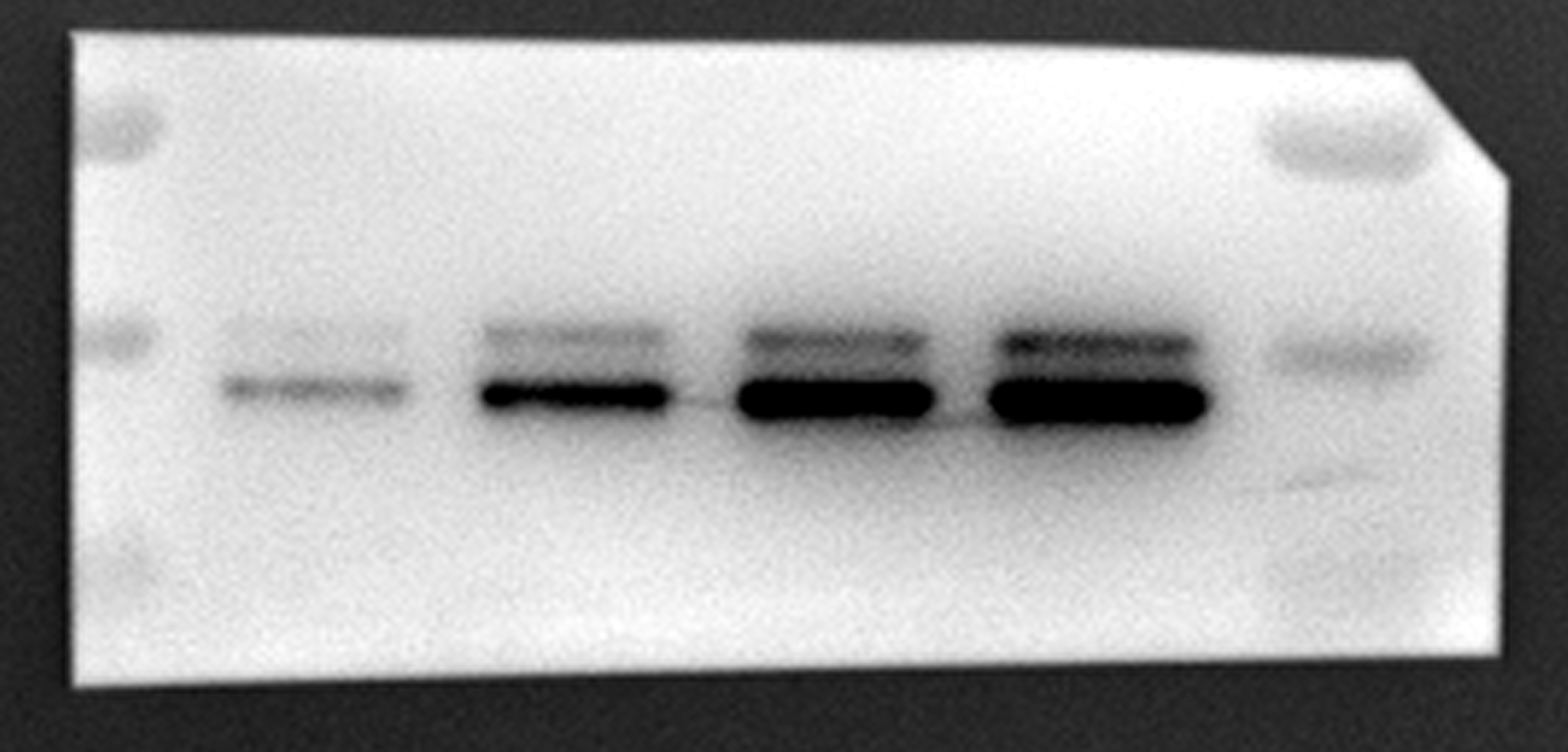

Supplement: Supplemental Material [file KBIE_A_2054195_SM0485.zip › supplementary/Figure1F_cleaved caspase3.tif]

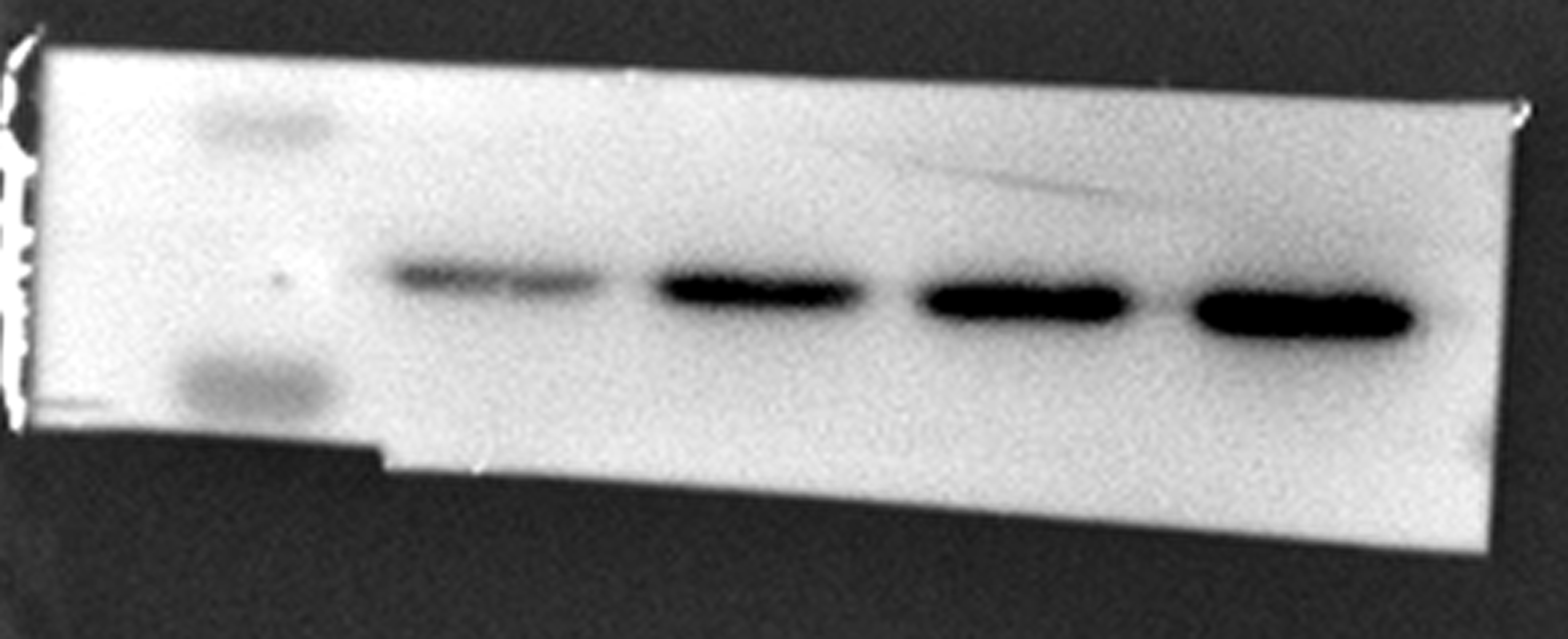

Supplement: Supplemental Material [file KBIE_A_2054195_SM0485.zip › supplementary/Figure1F_cleaved PARP.tif]

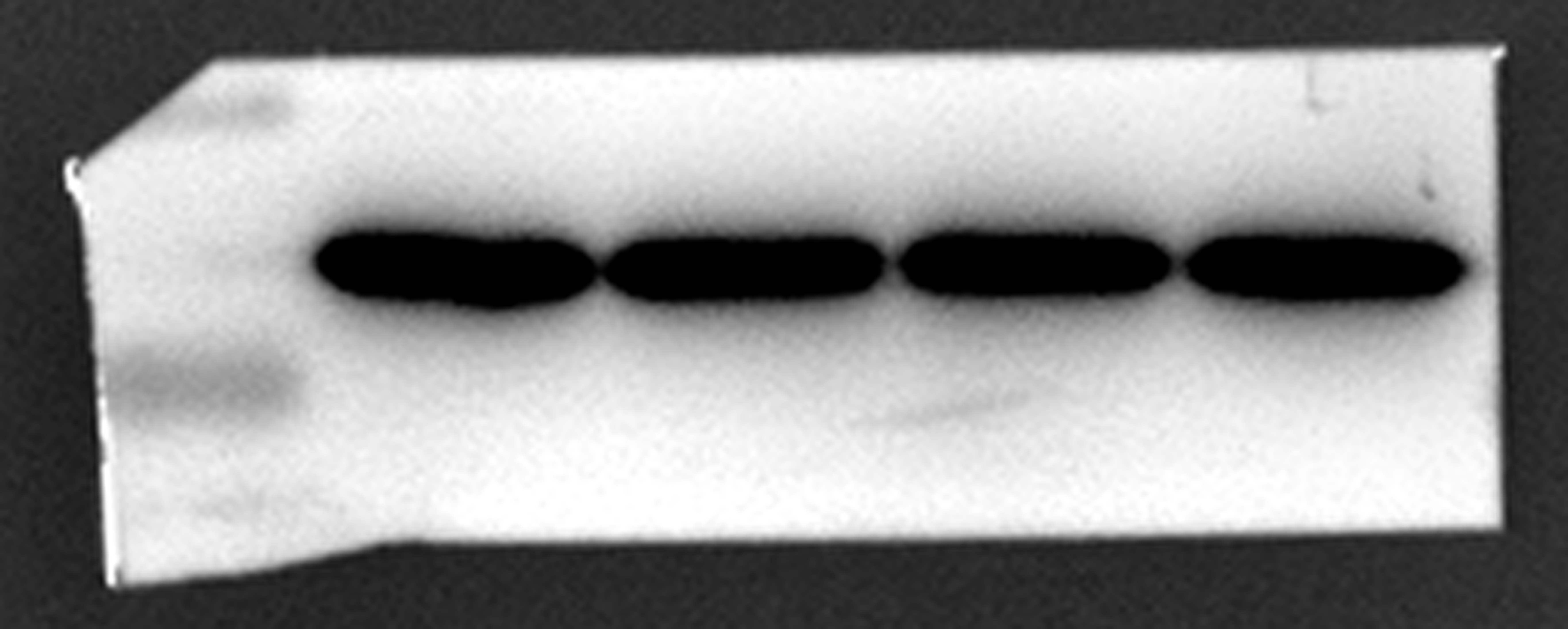

Supplement: Supplemental Material [file KBIE_A_2054195_SM0485.zip › supplementary/Figure1F_GAPDH.tif]

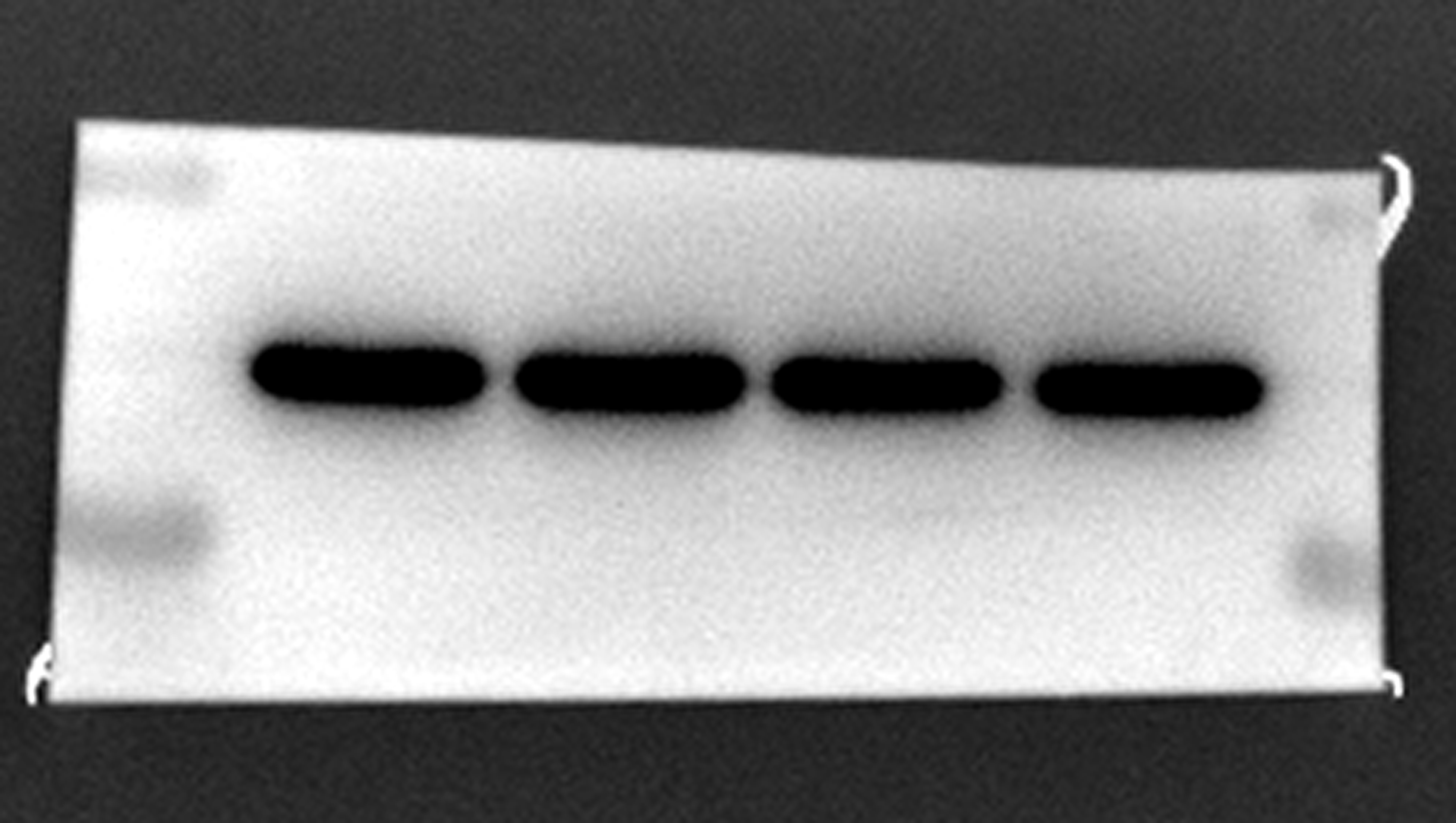

Supplement: Supplemental Material [file KBIE_A_2054195_SM0485.zip › supplementary/Figure1F_PARP.tif]

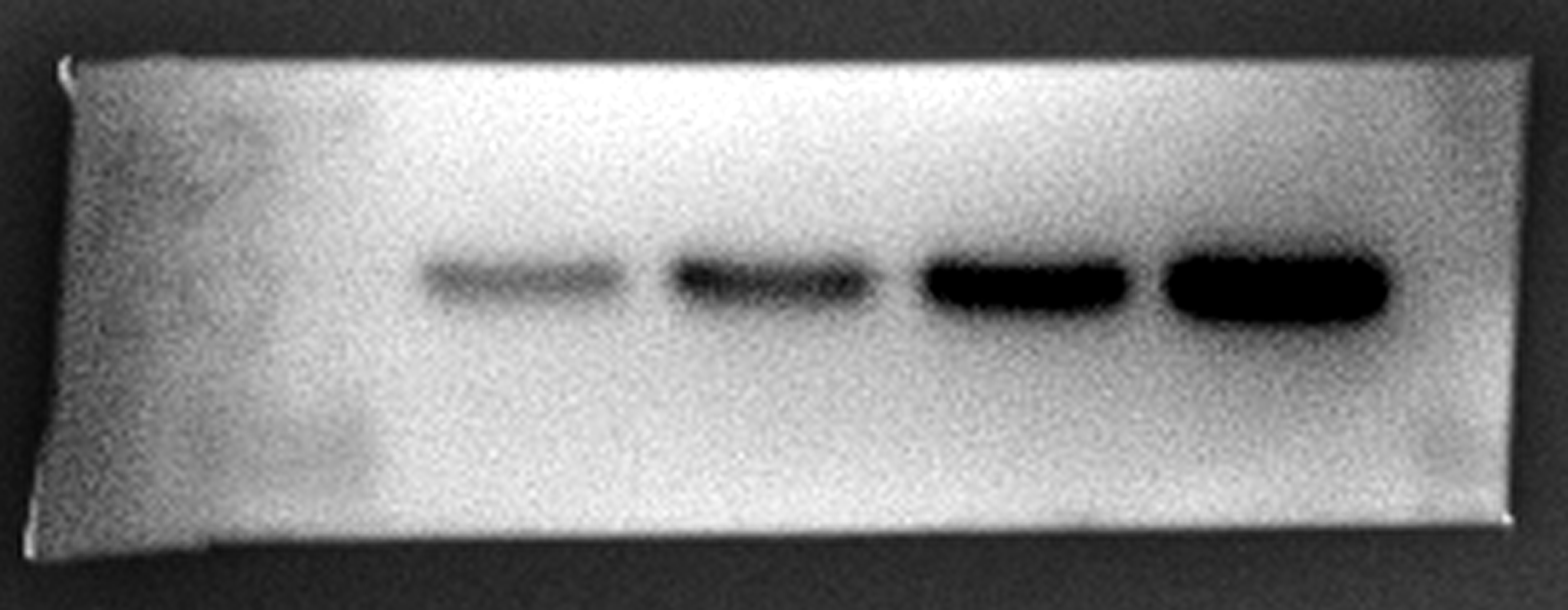

Supplement: Supplemental Material [file KBIE_A_2054195_SM0485.zip › supplementary/Figure2C_E_cadherin.tif]

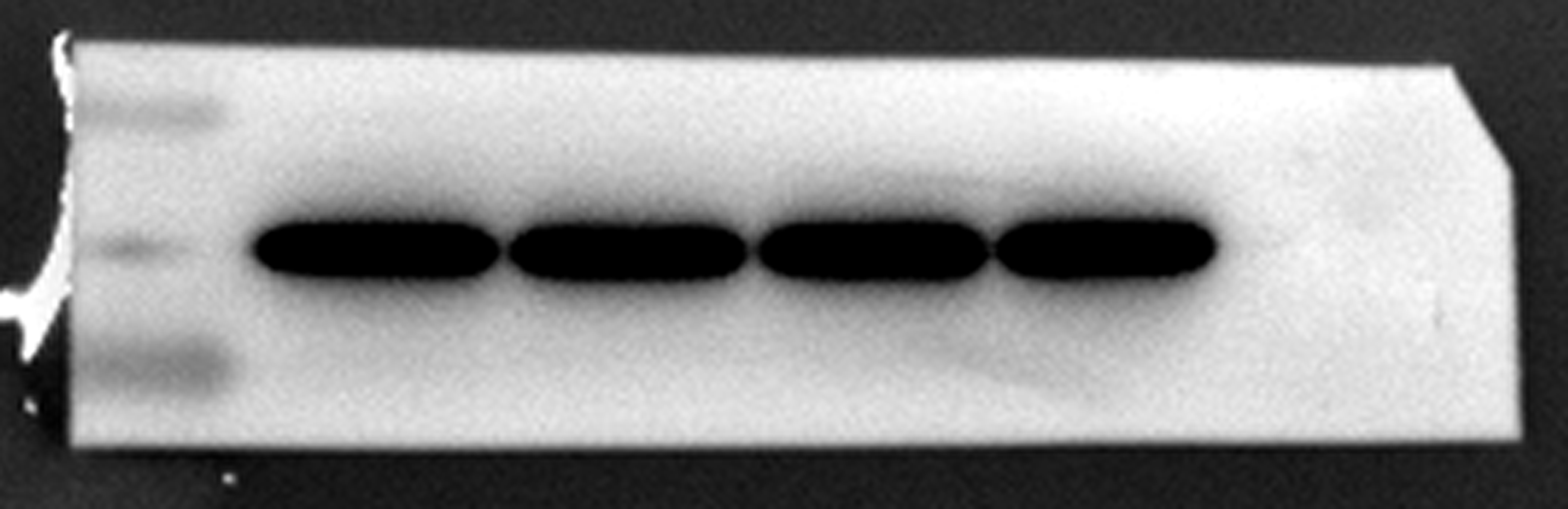

Supplement: Supplemental Material [file KBIE_A_2054195_SM0485.zip › supplementary/Figure2C_GAPDH.tif]

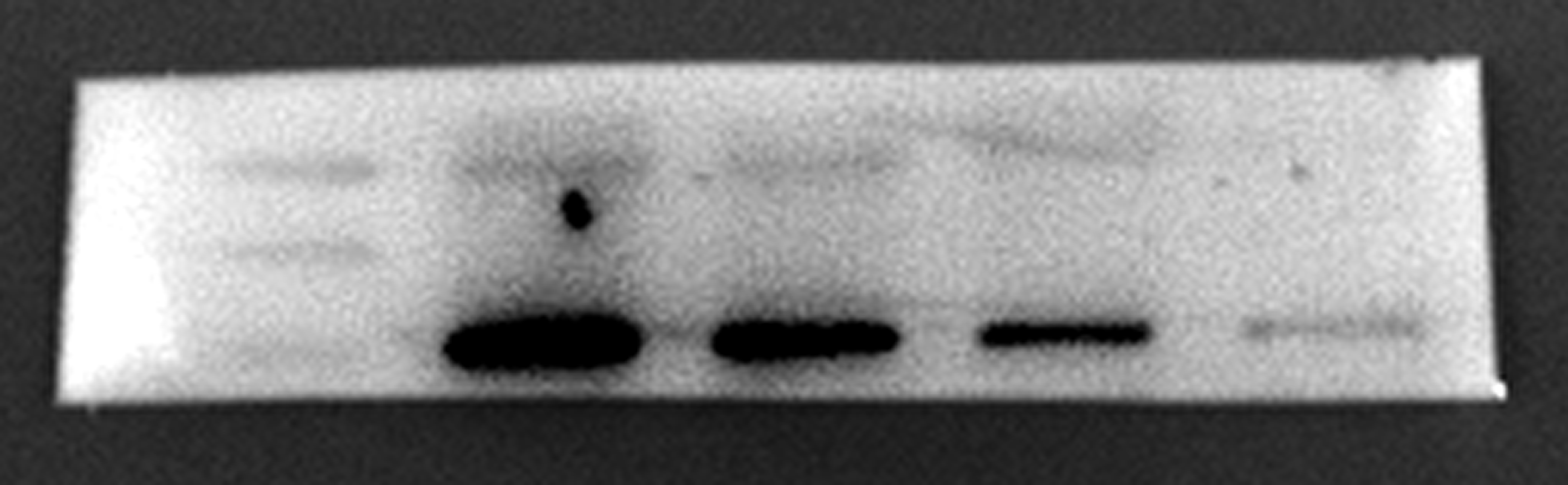

Supplement: Supplemental Material [file KBIE_A_2054195_SM0485.zip › supplementary/Figure2C_N_cadherin.tif]

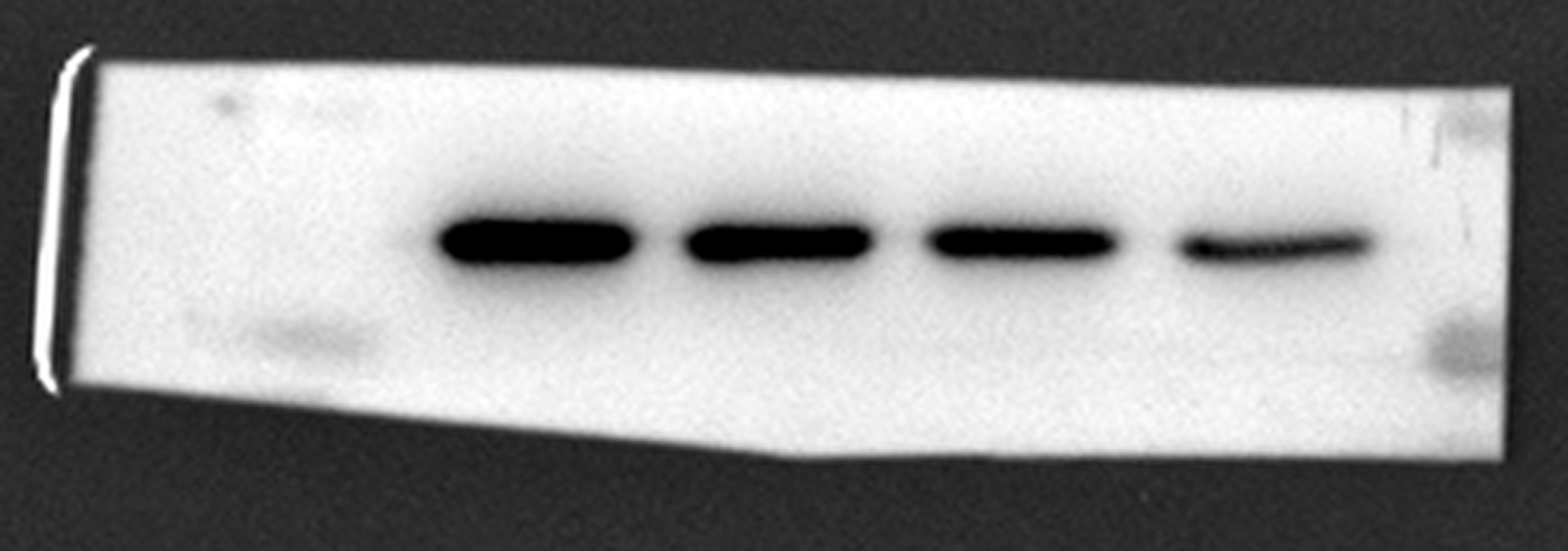

Supplement: Supplemental Material [file KBIE_A_2054195_SM0485.zip › supplementary/Figure2C_vimentin.tif]

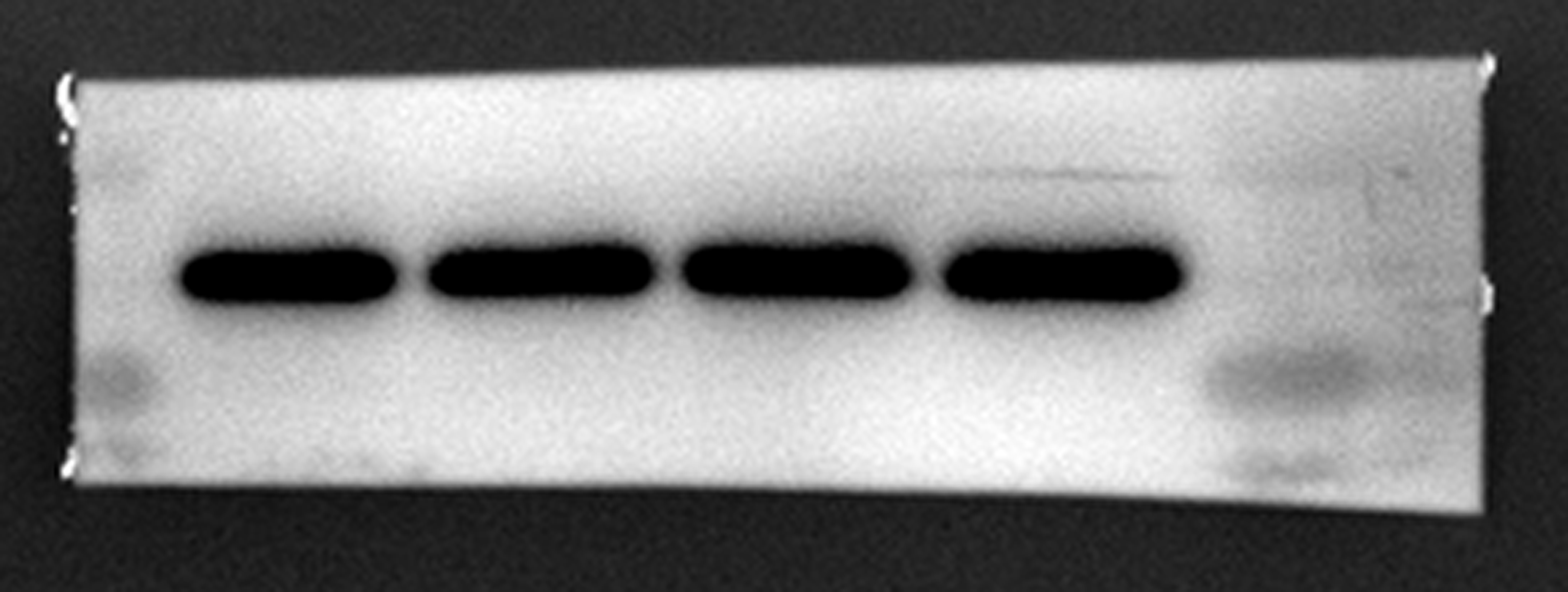

Supplement: Supplemental Material [file KBIE_A_2054195_SM0485.zip › supplementary/Figure3B_GAPDH.tif]

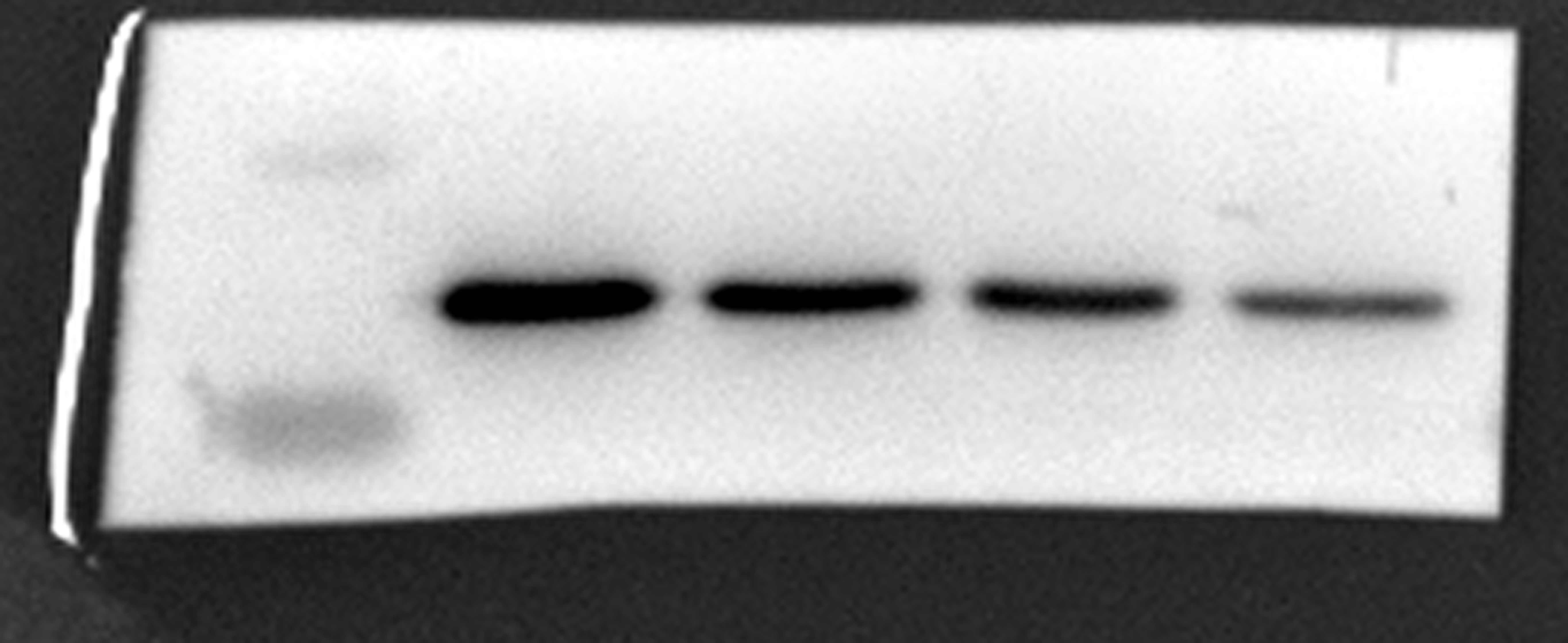

Supplement: Supplemental Material [file KBIE_A_2054195_SM0485.zip › supplementary/Figure3B_PLK4.tif]

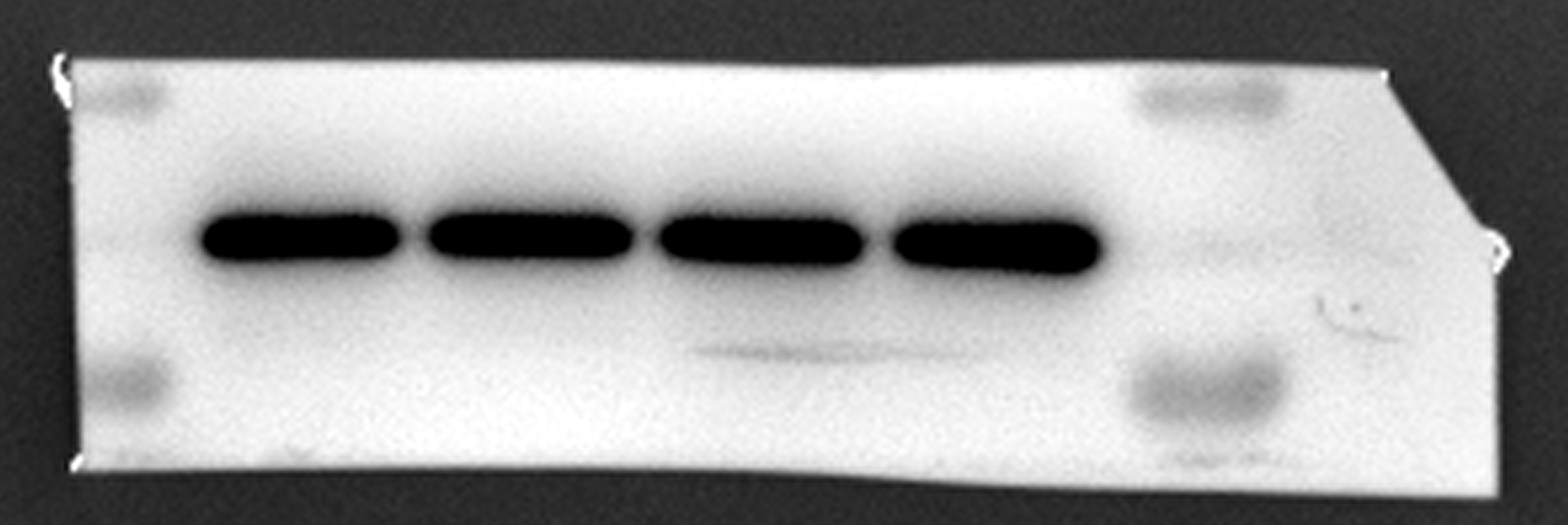

Supplement: Supplemental Material [file KBIE_A_2054195_SM0485.zip › supplementary/Figure3C_AKT.tif]

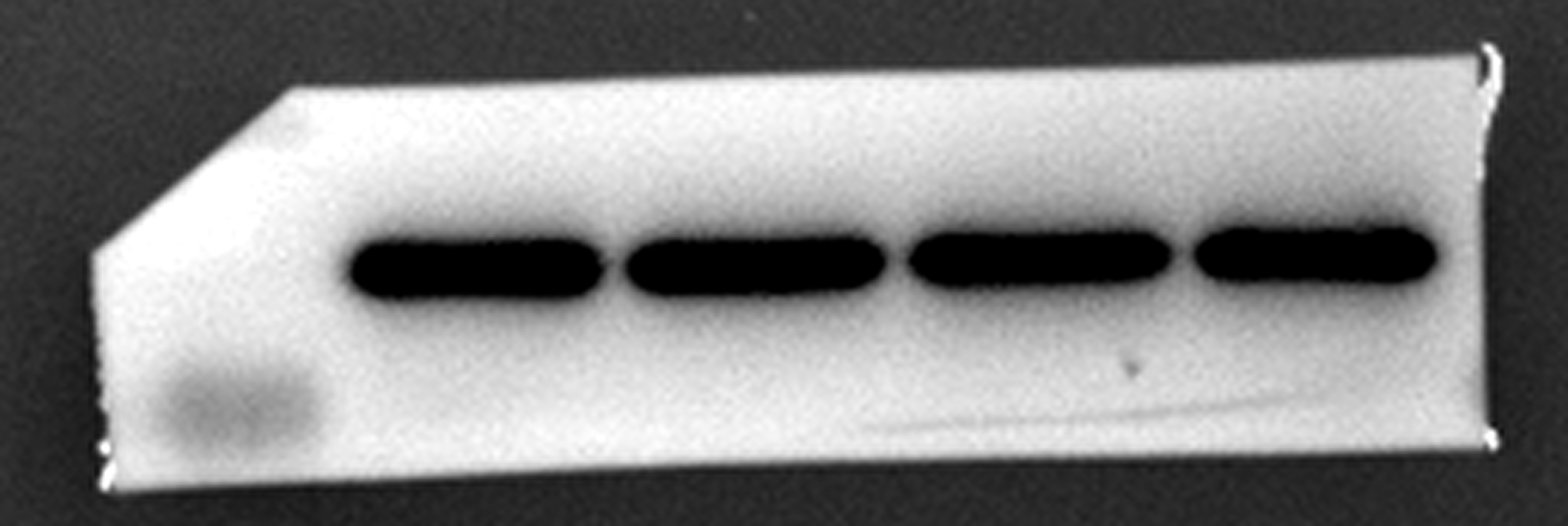

Supplement: Supplemental Material [file KBIE_A_2054195_SM0485.zip › supplementary/Figure3C_GAPDH.tif]

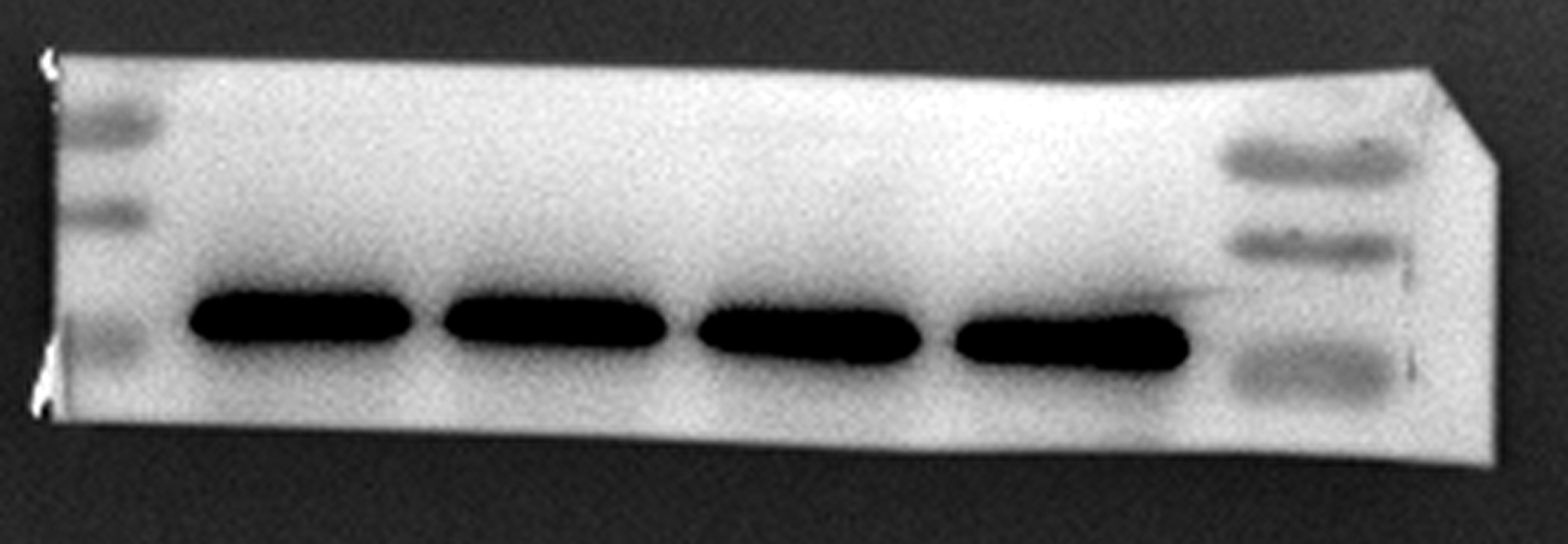

Supplement: Supplemental Material [file KBIE_A_2054195_SM0485.zip › supplementary/Figure3C_PI3K.tif]

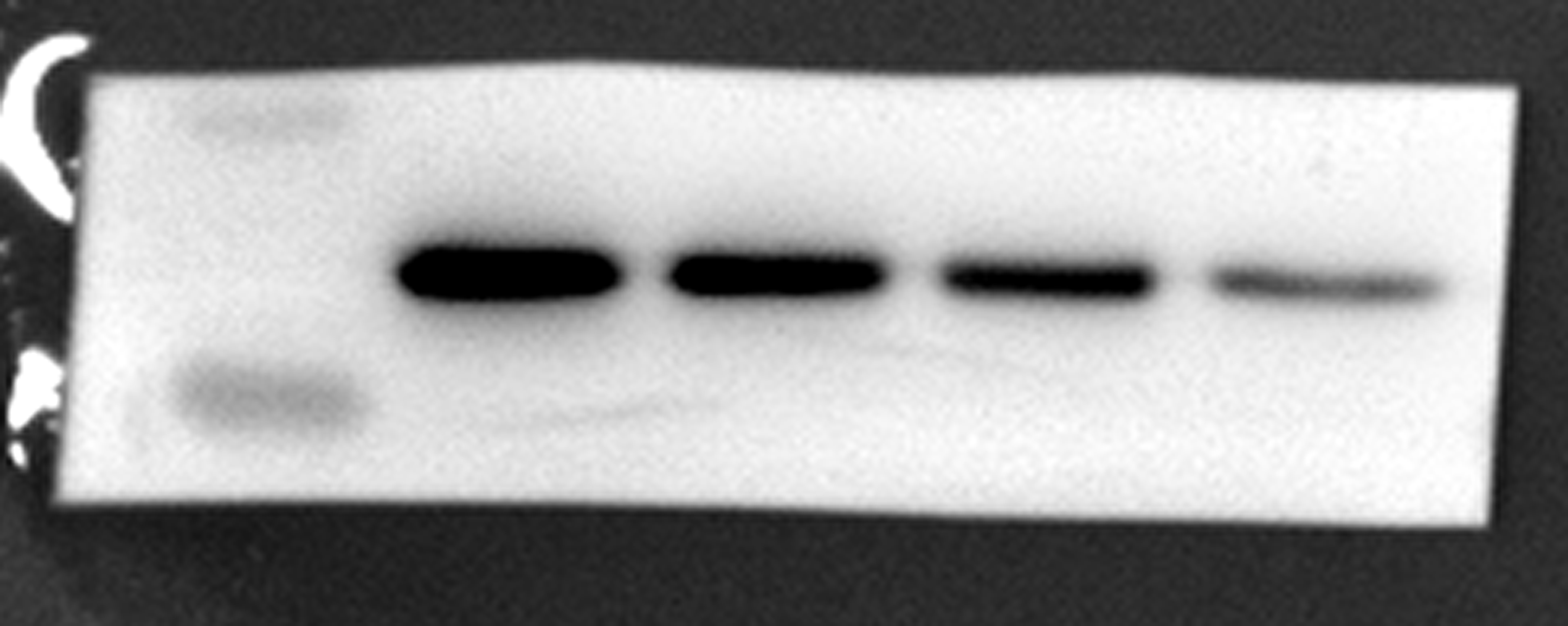

Supplement: Supplemental Material [file KBIE_A_2054195_SM0485.zip › supplementary/Figure3C_p_AKT.tif]

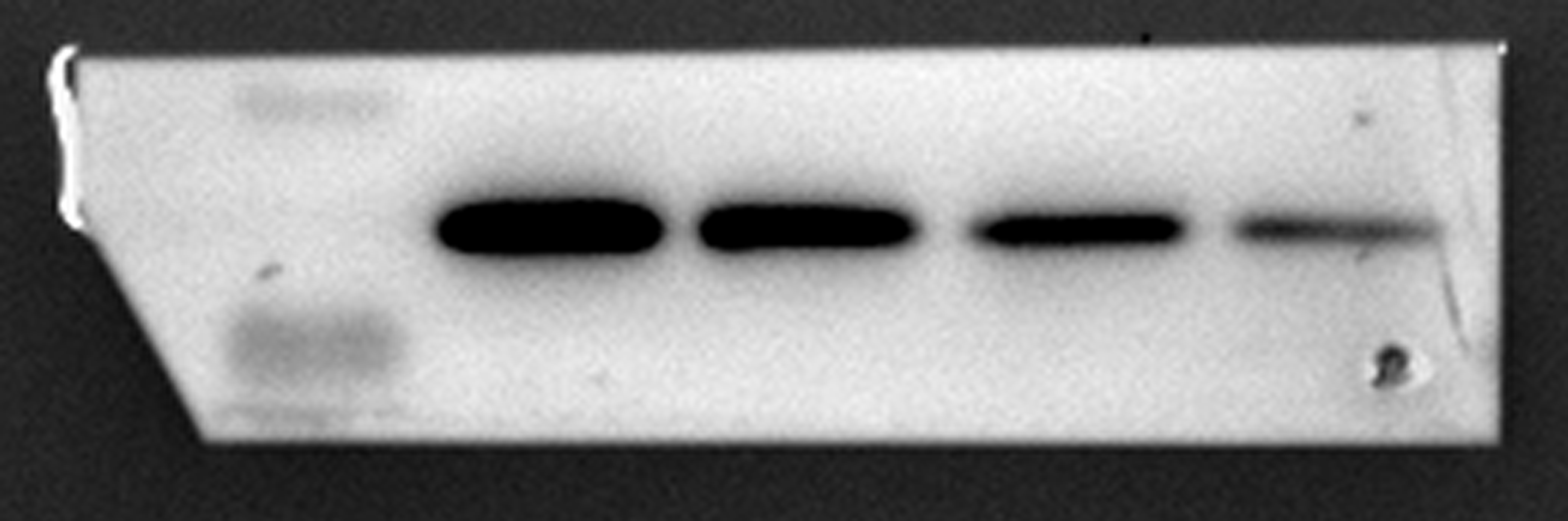

Supplement: Supplemental Material [file KBIE_A_2054195_SM0485.zip › supplementary/Figure3C_p_PI3K.tif]

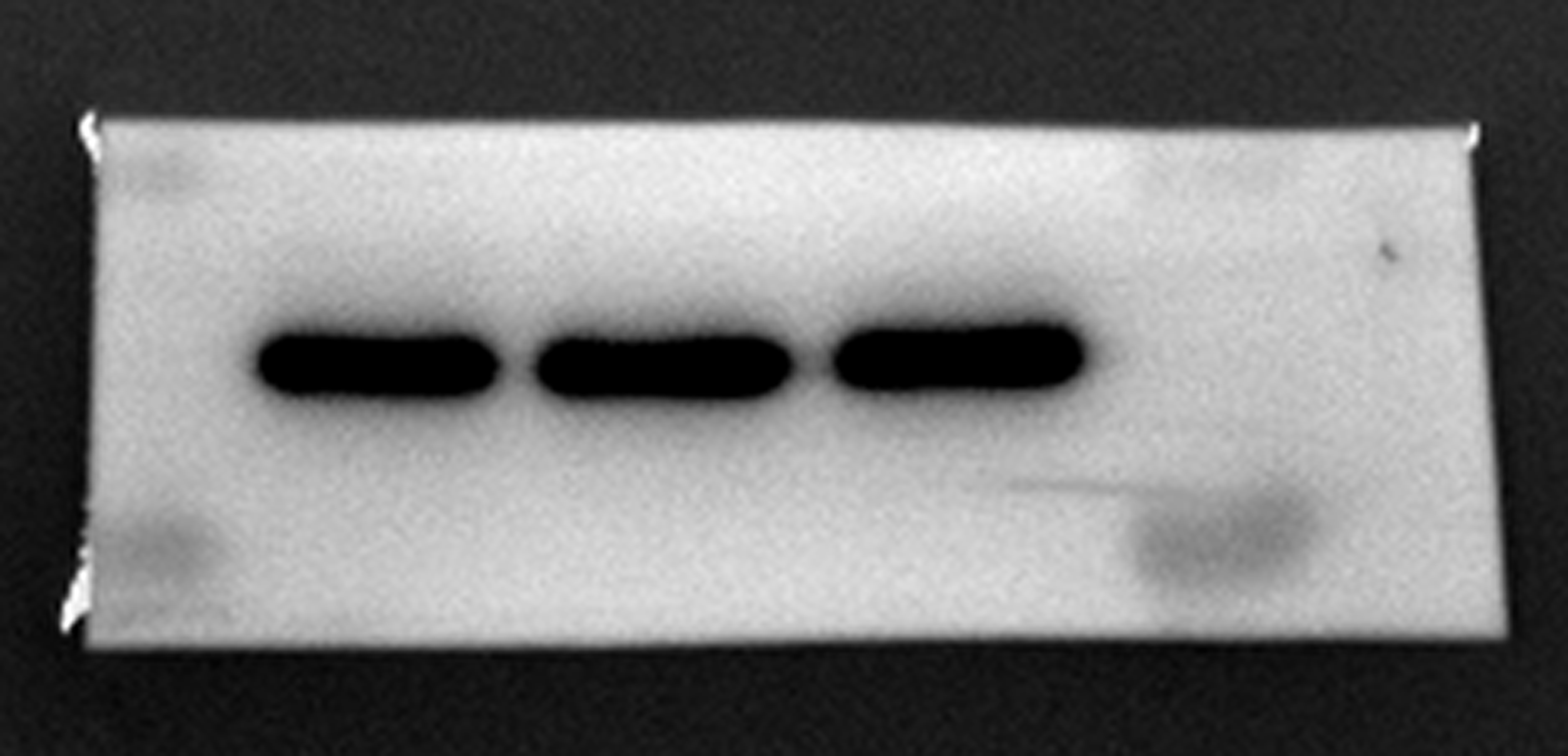

Supplement: Supplemental Material [file KBIE_A_2054195_SM0485.zip › supplementary/Figure4B_GAPDH.tif]

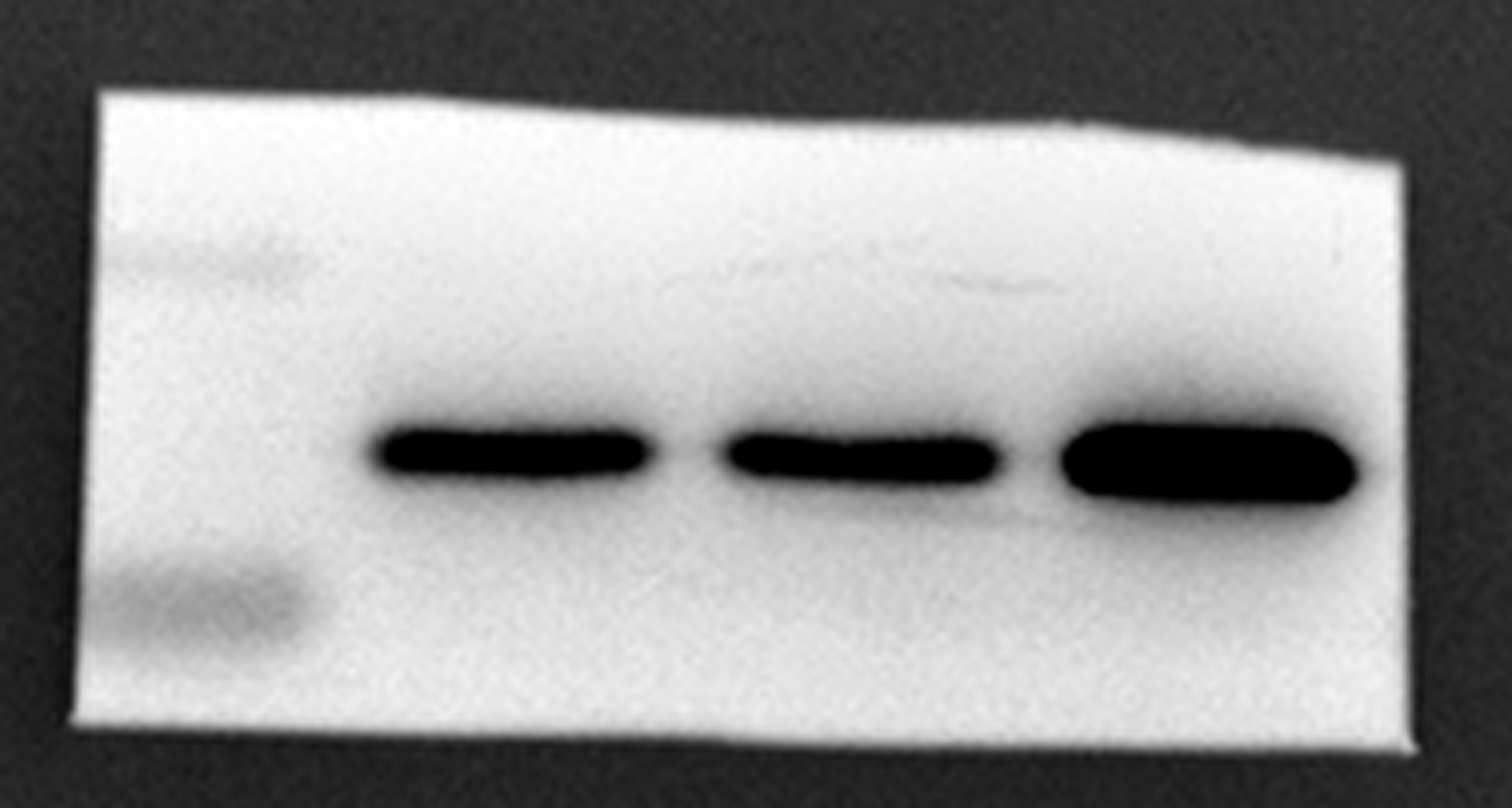

Supplement: Supplemental Material [file KBIE_A_2054195_SM0485.zip › supplementary/Figure4B_PLK4.tif]

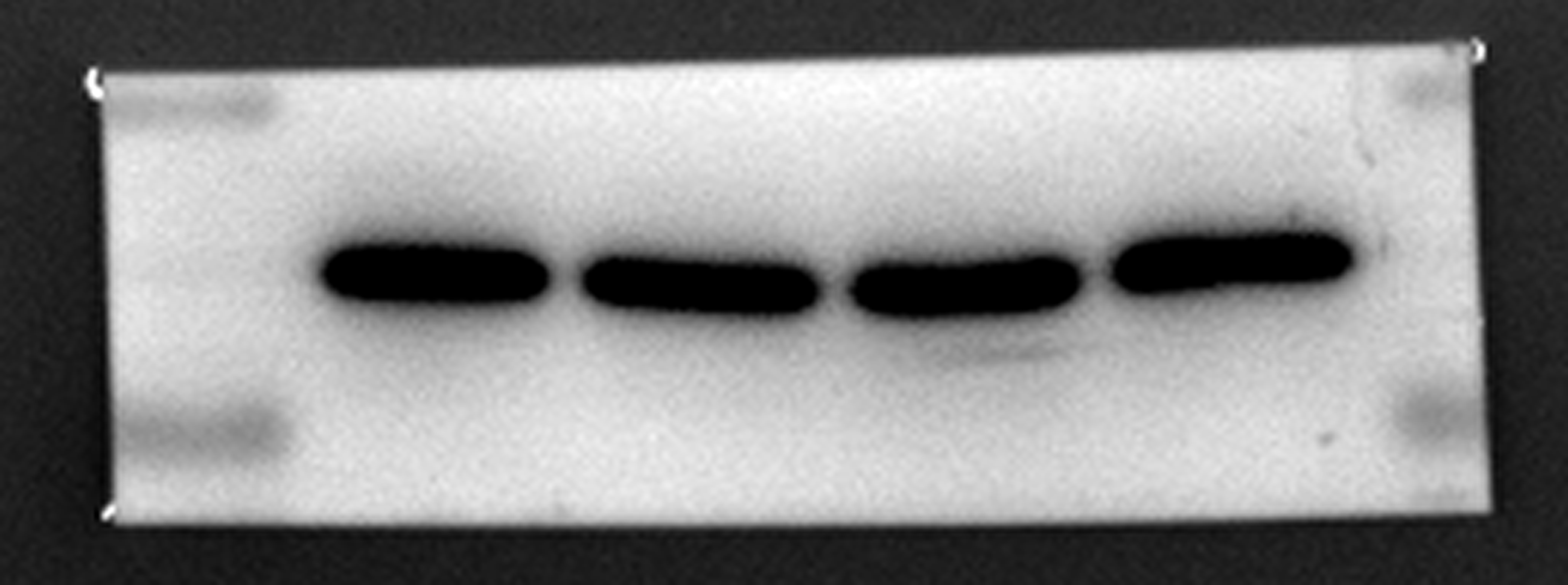

Supplement: Supplemental Material [file KBIE_A_2054195_SM0485.zip › supplementary/Figure4C_AKT.tif]

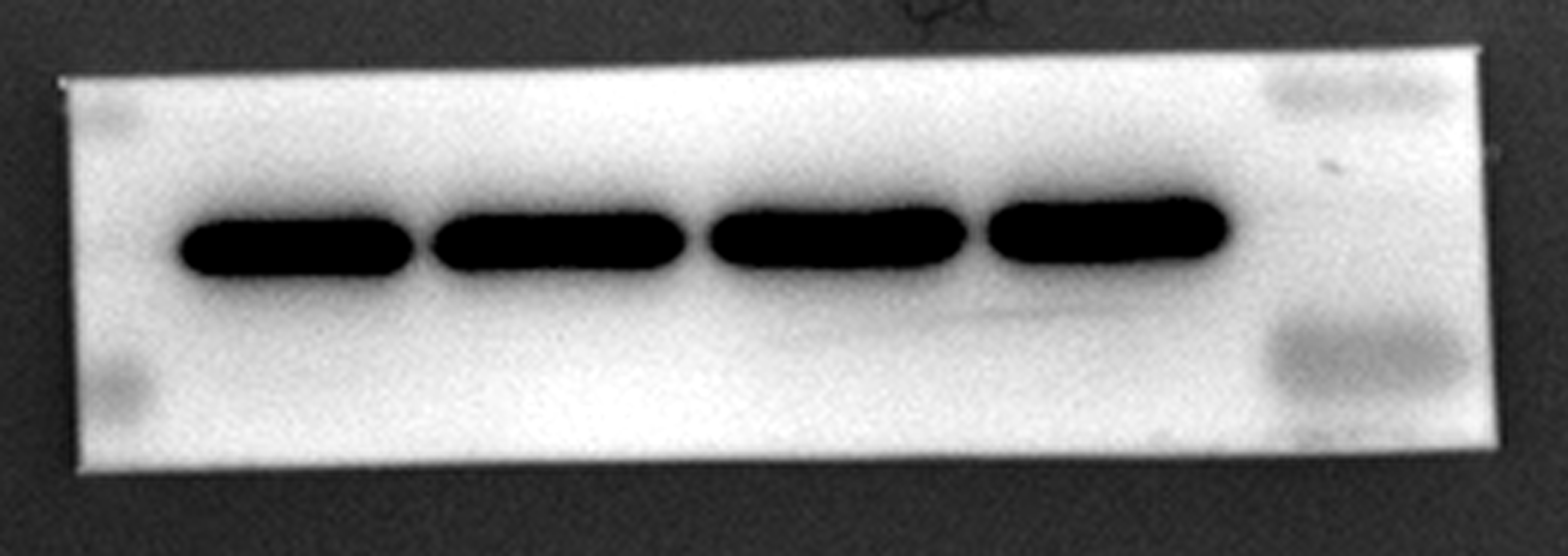

Supplement: Supplemental Material [file KBIE_A_2054195_SM0485.zip › supplementary/Figure4C_GAPDH.tif]

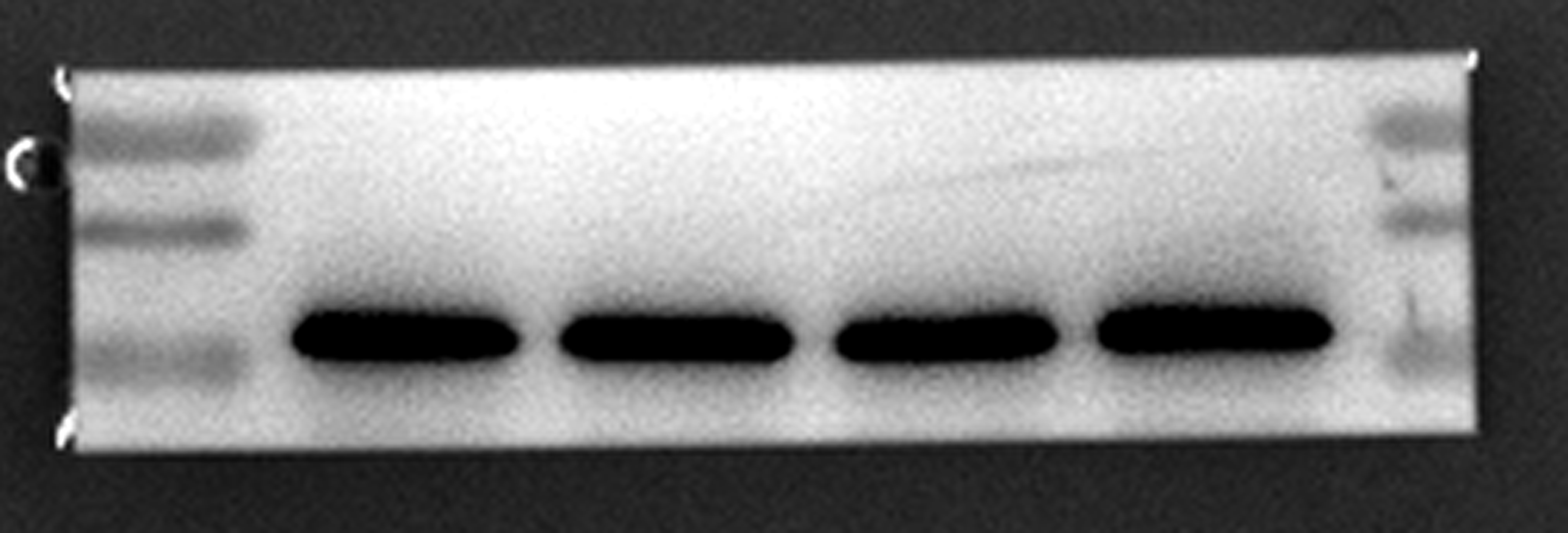

Supplement: Supplemental Material [file KBIE_A_2054195_SM0485.zip › supplementary/Figure4C_PI3K.tif]

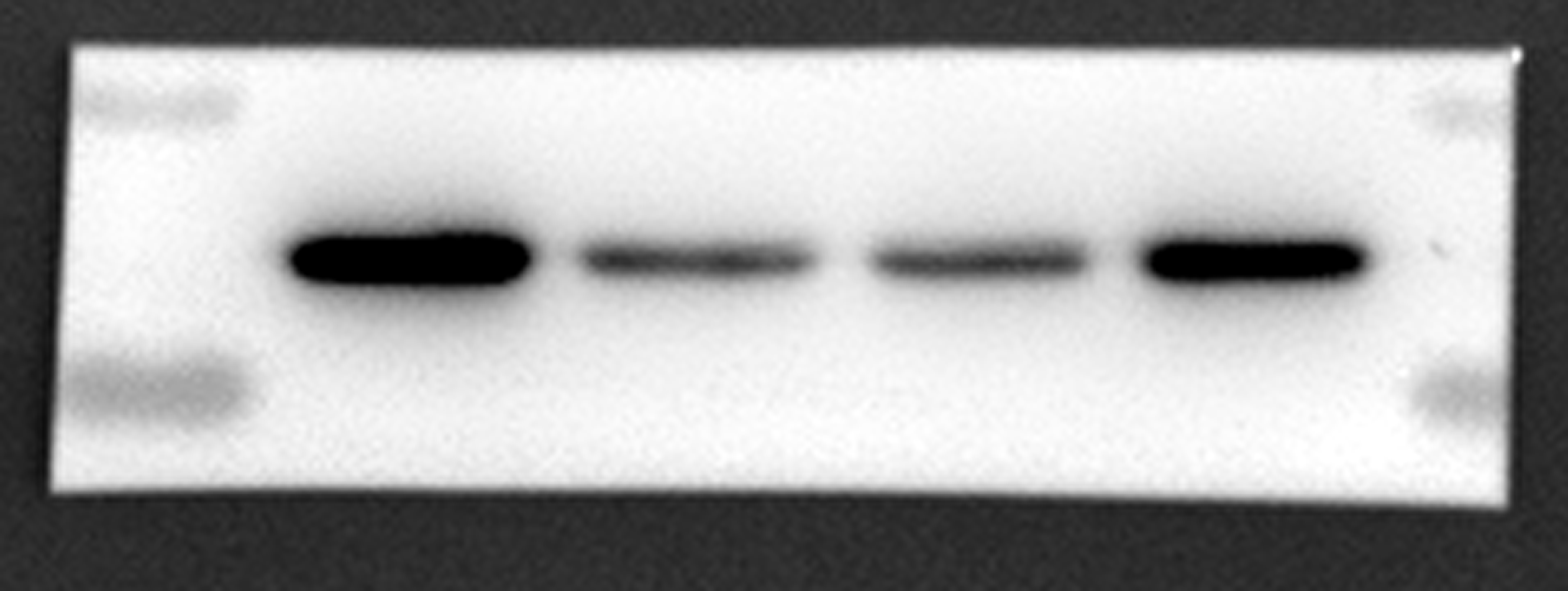

Supplement: Supplemental Material [file KBIE_A_2054195_SM0485.zip › supplementary/Figure4C_p_AKT.tif]

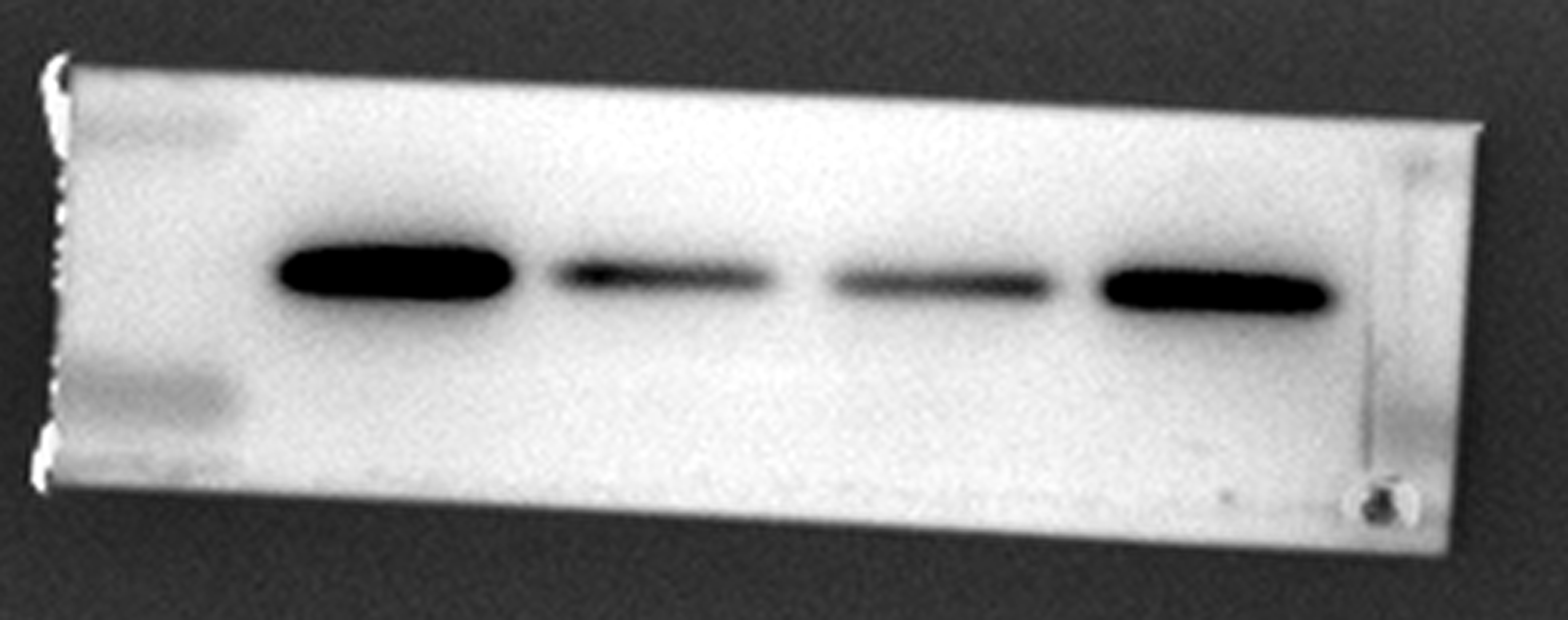

Supplement: Supplemental Material [file KBIE_A_2054195_SM0485.zip › supplementary/Figure4C_p_PI3K.tif]

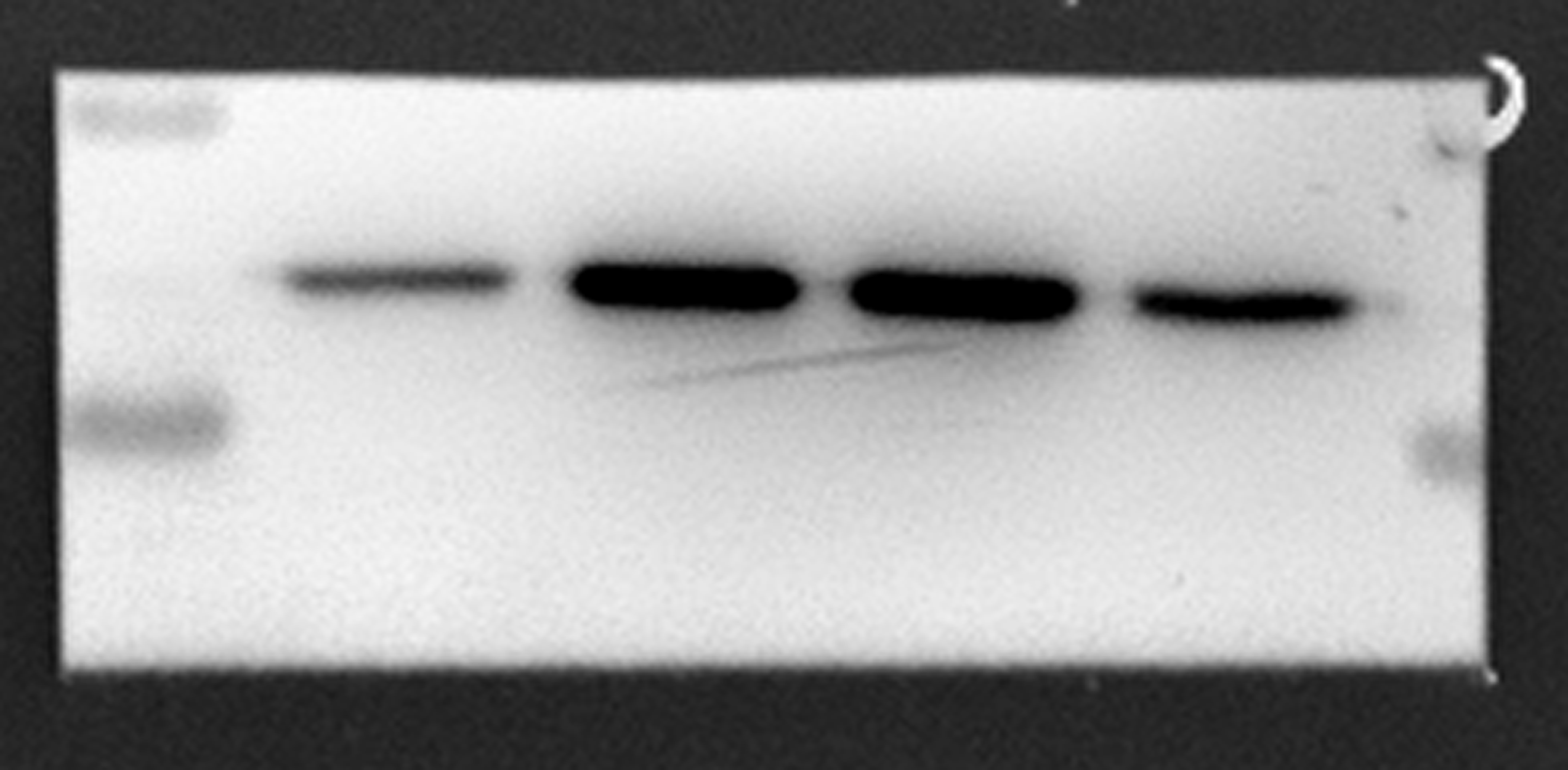

Supplement: Supplemental Material [file KBIE_A_2054195_SM0485.zip › supplementary/Figure5B_Bax.tif]

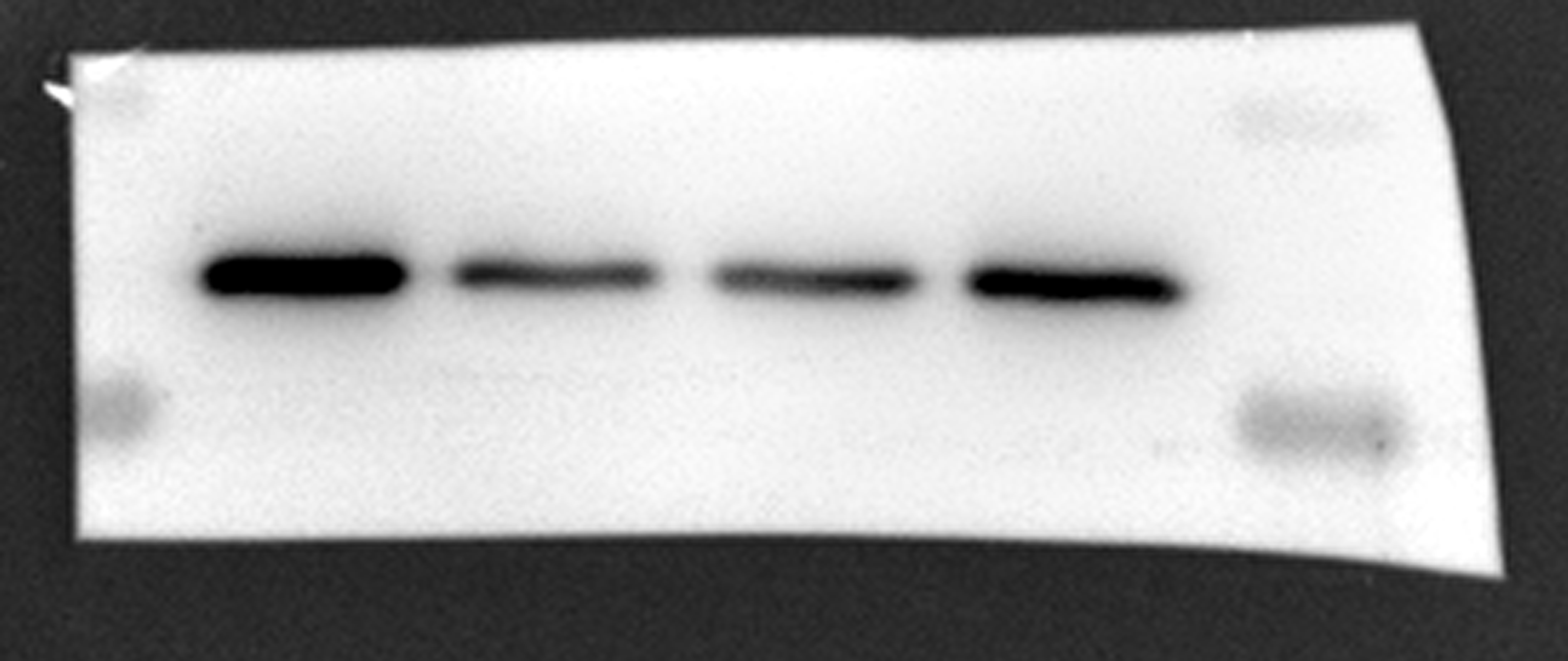

Supplement: Supplemental Material [file KBIE_A_2054195_SM0485.zip › supplementary/Figure5B_Bcl_2.tif]

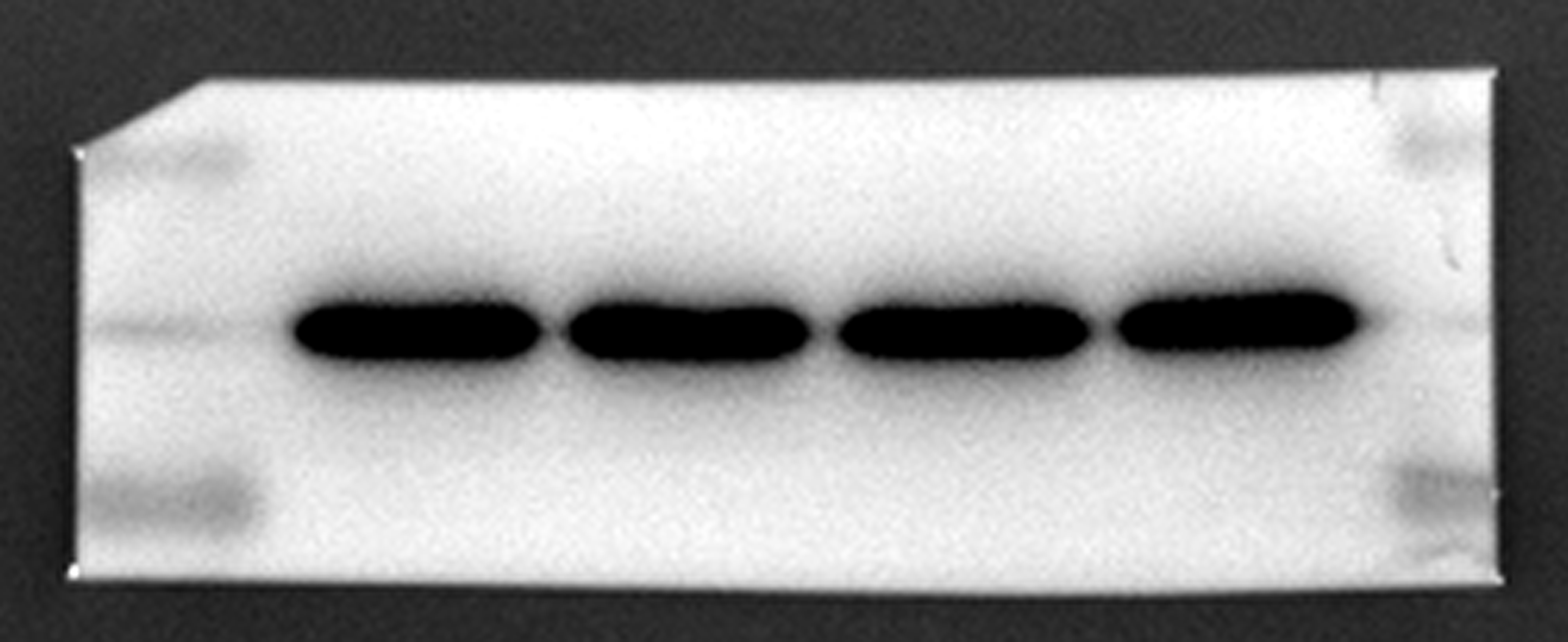

Supplement: Supplemental Material [file KBIE_A_2054195_SM0485.zip › supplementary/Figure5B_caspase3.tif]

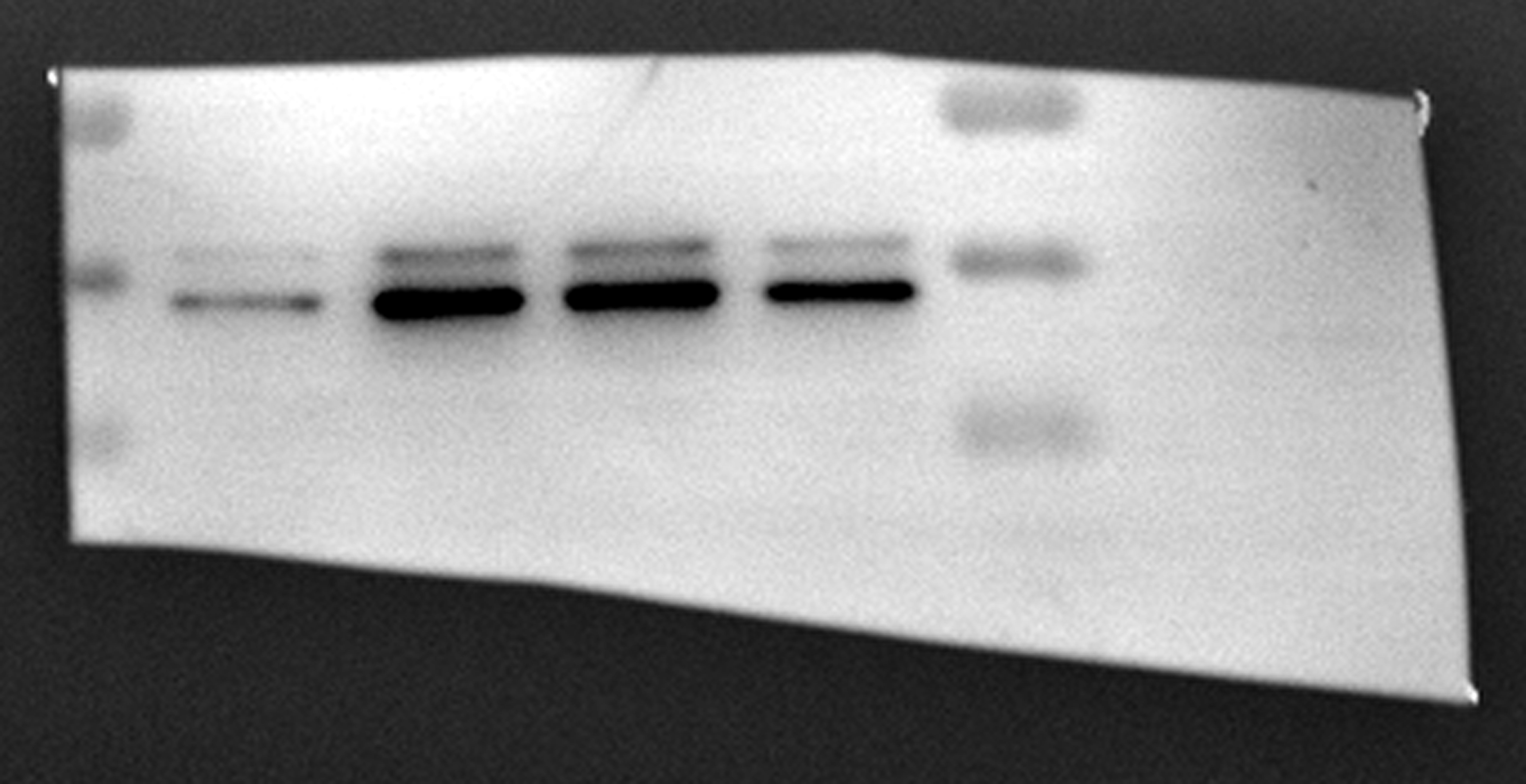

Supplement: Supplemental Material [file KBIE_A_2054195_SM0485.zip › supplementary/Figure5B_cleaved caspase3.tif]

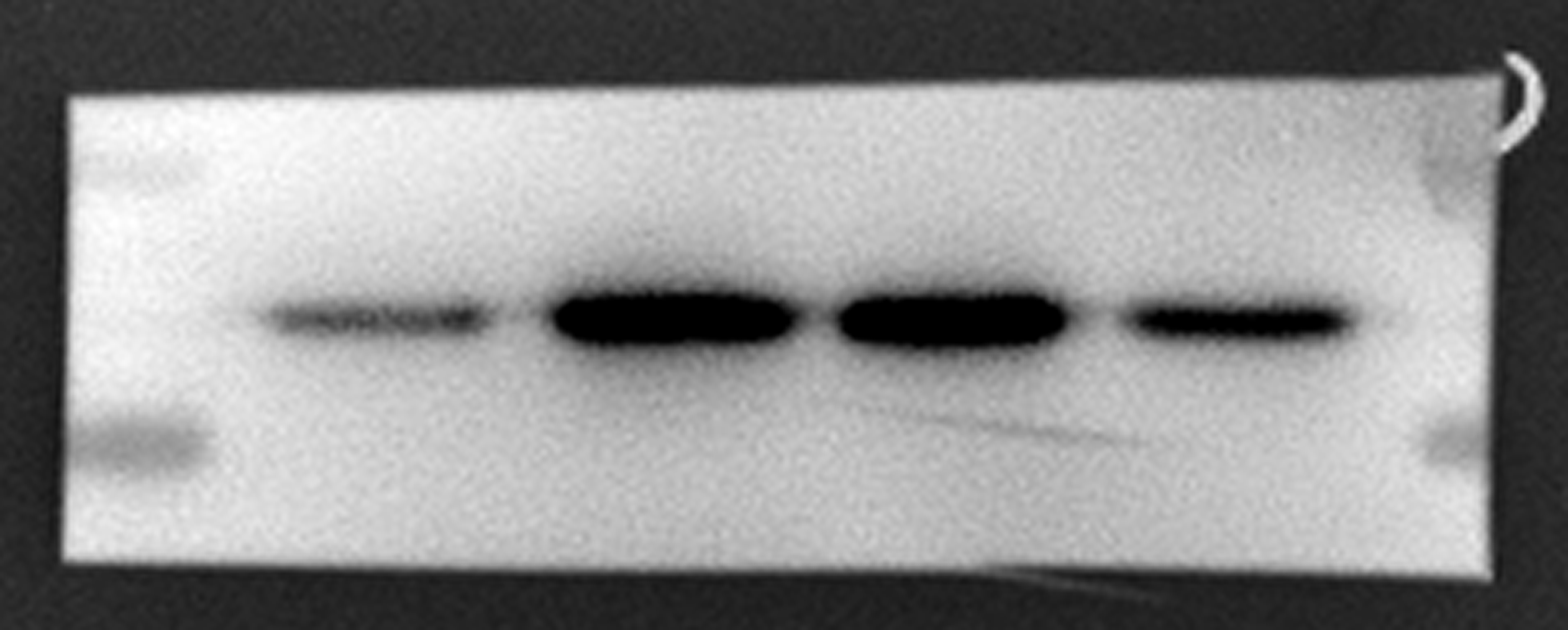

Supplement: Supplemental Material [file KBIE_A_2054195_SM0485.zip › supplementary/Figure5B_cleaved PARP.tif]

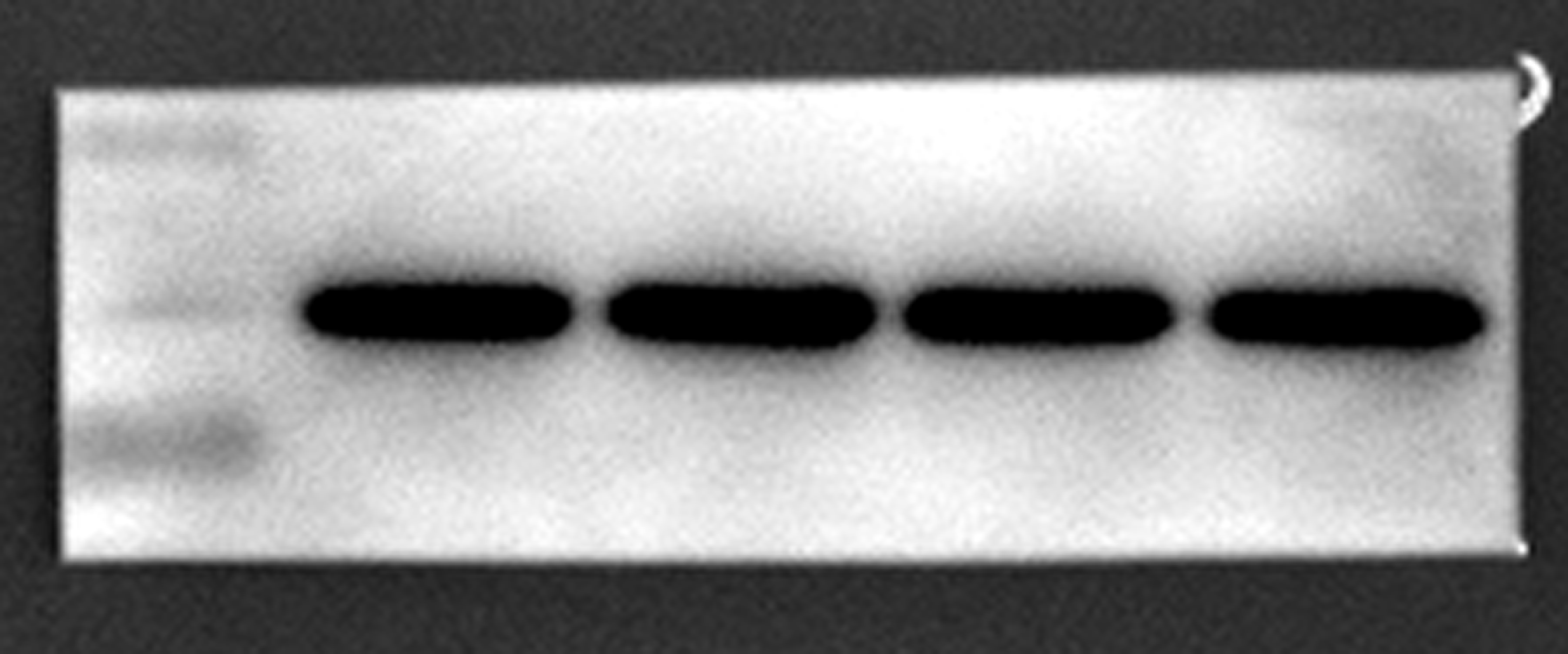

Supplement: Supplemental Material [file KBIE_A_2054195_SM0485.zip › supplementary/Figure5B_GAPDH.tif]

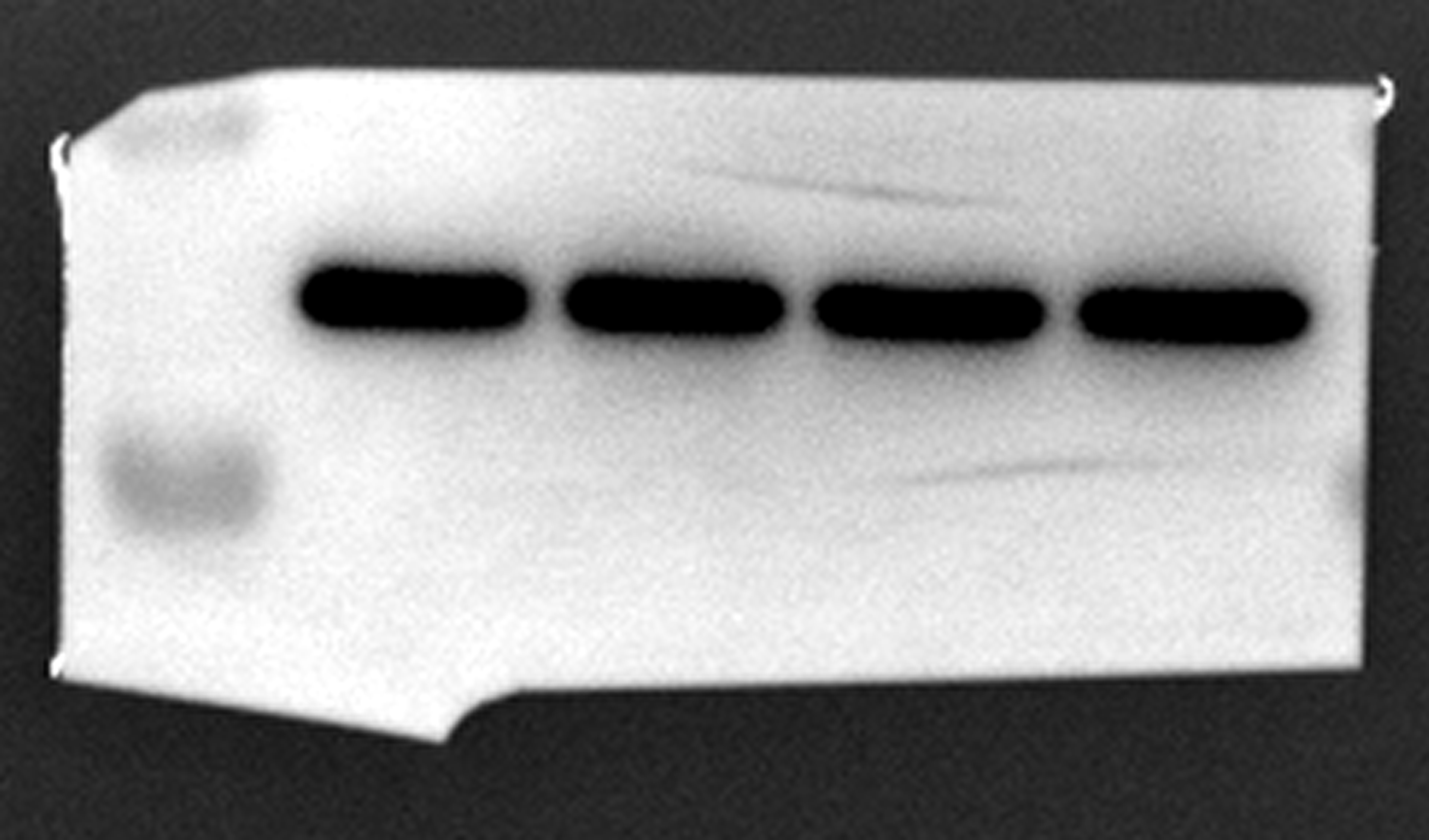

Supplement: Supplemental Material [file KBIE_A_2054195_SM0485.zip › supplementary/Figure5B_PARP.tif]

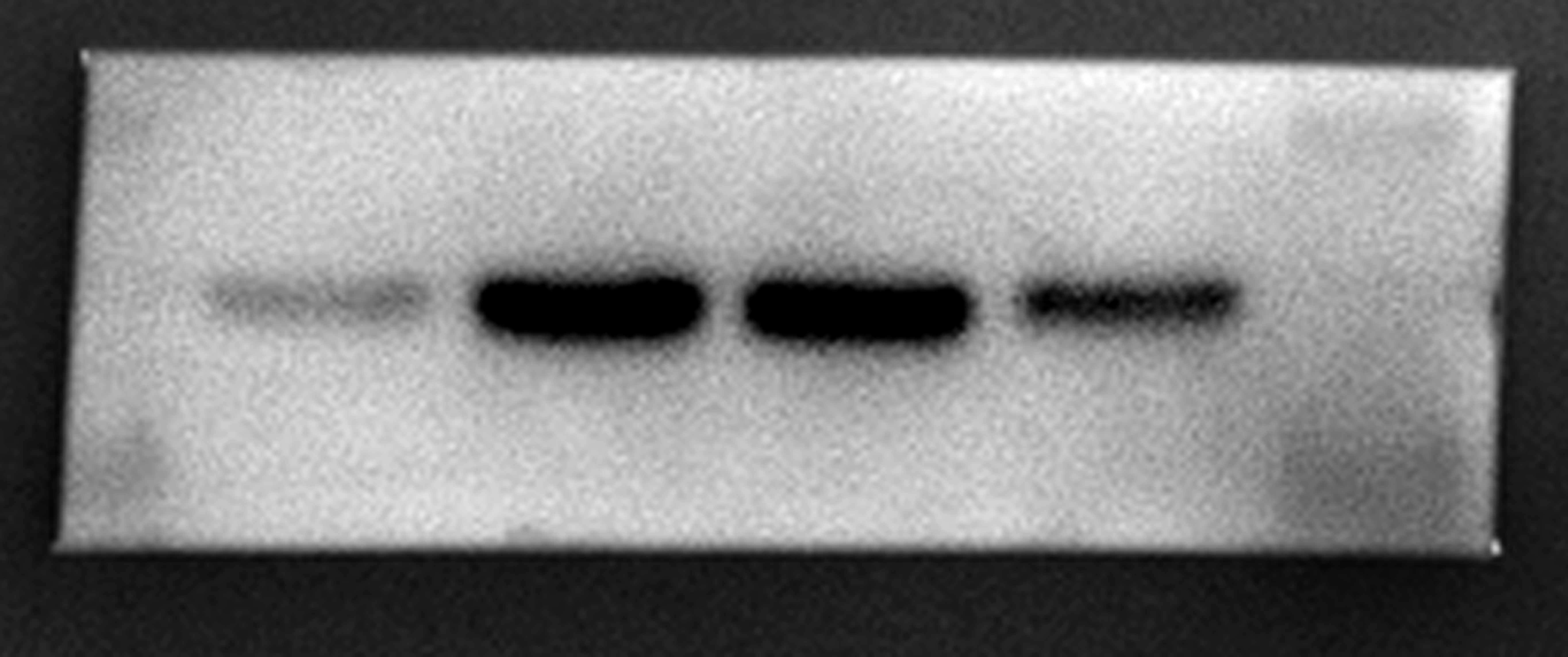

Supplement: Supplemental Material [file KBIE_A_2054195_SM0485.zip › supplementary/Figure6C_E_cadherin.tif]

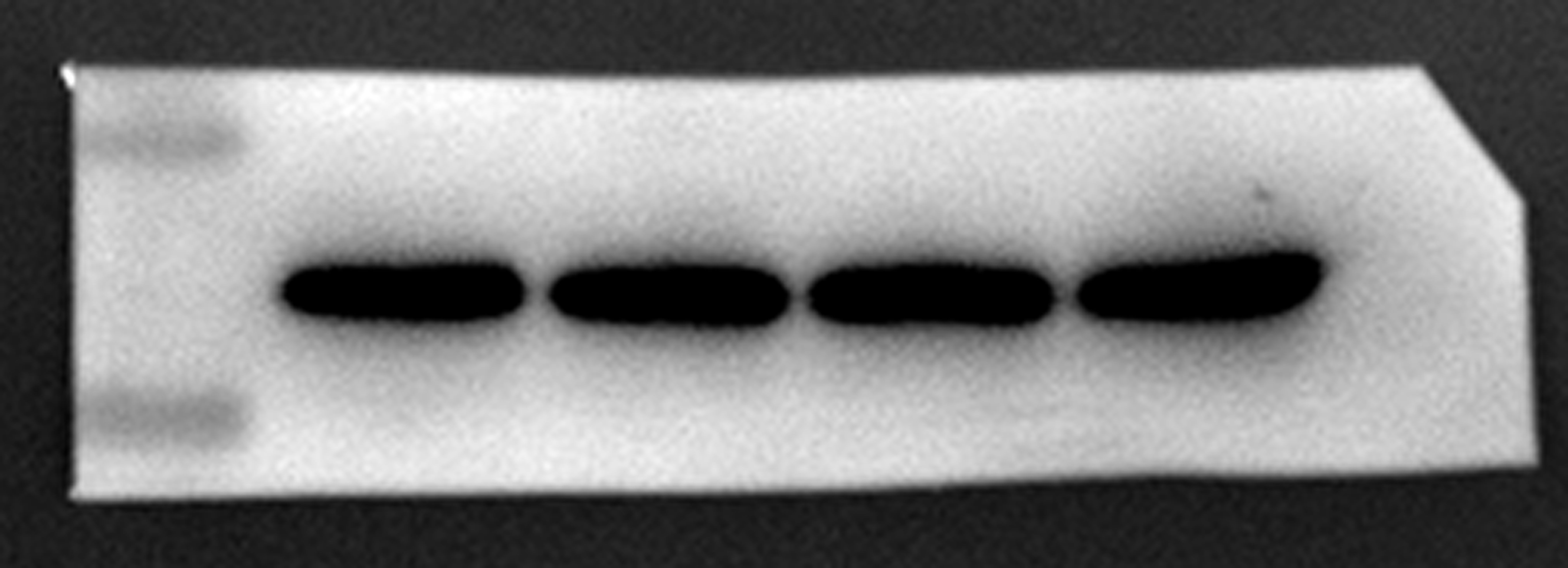

Supplement: Supplemental Material [file KBIE_A_2054195_SM0485.zip › supplementary/Figure6C_GAPDH.tif]

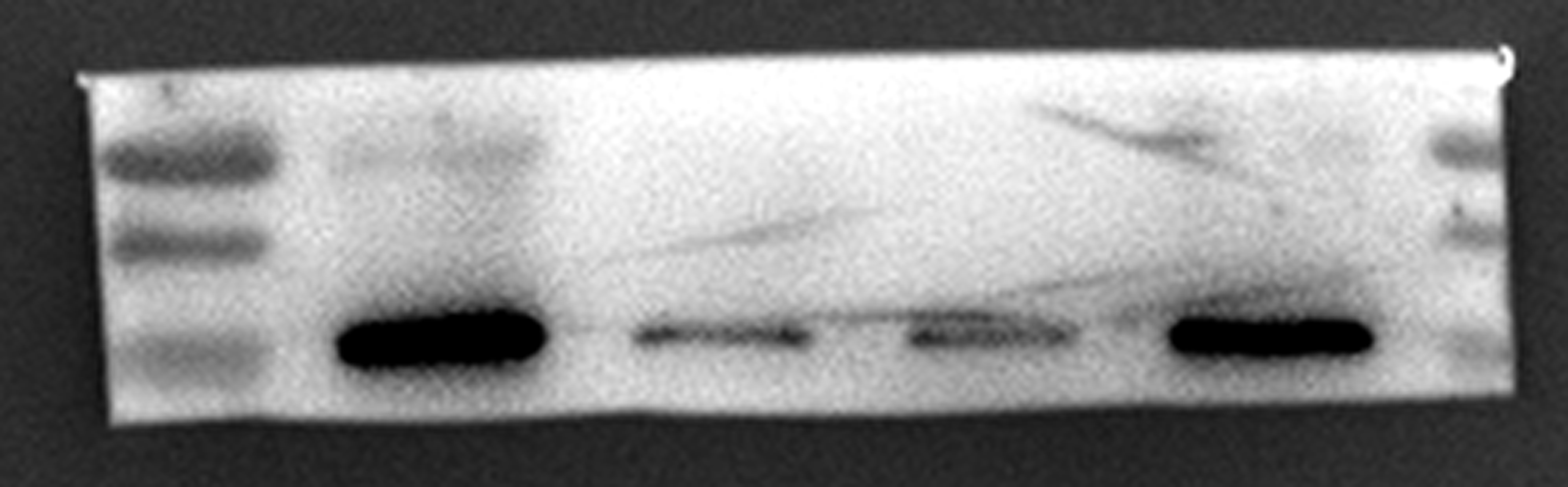

Supplement: Supplemental Material [file KBIE_A_2054195_SM0485.zip › supplementary/Figure6C_N_cadherin.tif]

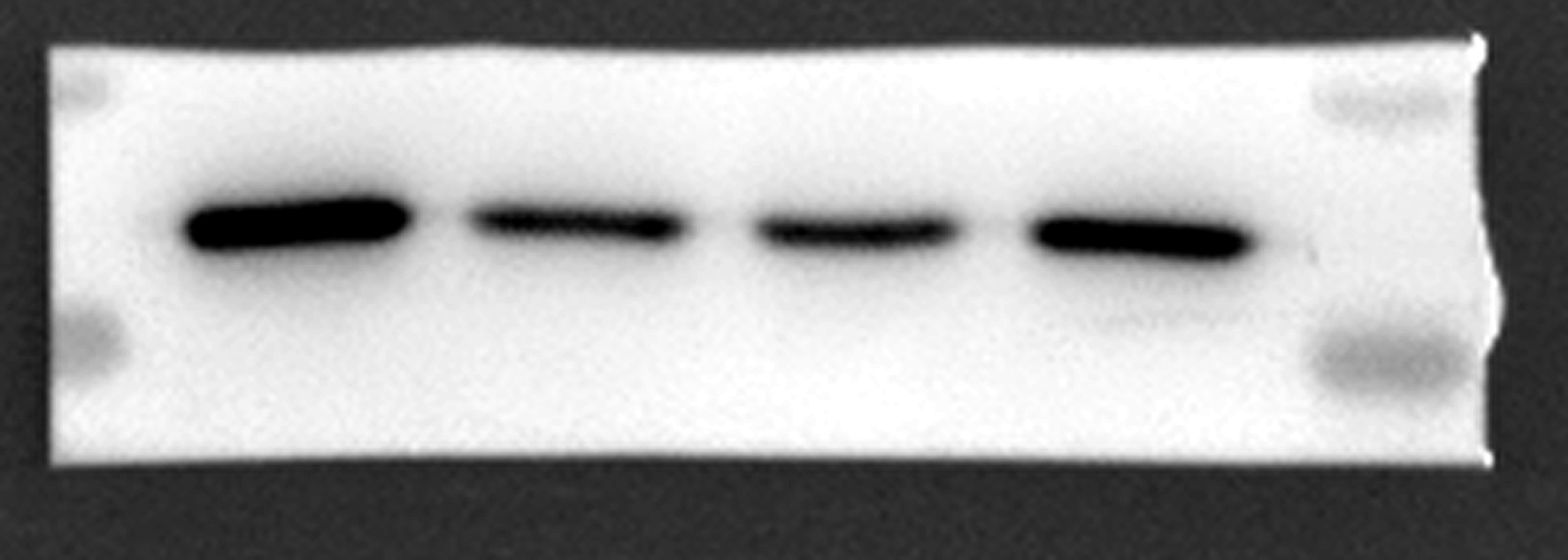

Supplement: Supplemental Material [file KBIE_A_2054195_SM0485.zip › supplementary/Figure6C_vimentin.tif]
